# Pericyte-Mediated Tissue Repair through PDGFRβ Promotes Peri-Infarct Astrogliosis, Oligodendrogenesis, and Functional Recovery after Acute Ischemic Stroke

**DOI:** 10.1523/ENEURO.0474-19.2020

**Published:** 2020-03-03

**Authors:** Tomoya Shibahara, Tetsuro Ago, Kuniyuki Nakamura, Masaki Tachibana, Yoji Yoshikawa, Motohiro Komori, Kei Yamanaka, Yoshinobu Wakisaka, Takanari Kitazono

**Affiliations:** Department of Medicine and Clinical Science, Graduate School of Medical Sciences, Kyushu University, Fukuoka 812-8582, Japan

**Keywords:** astrocyte, neurorestoration, oligodendrogenesis, pericyte, platelet-derived growth factor receptor β, repair

## Abstract

Post-stroke functional recovery can occur spontaneously during the subacute phase; however, how post-stroke fibrotic repair affects functional recovery is highly debated. Platelet-derived growth factor receptor β (PDGFRβ)-expressing pericytes are responsible for post-stroke fibrotic repair within infarct areas; therefore, we examined peri-infarct neural reorganization and functional recovery after permanent middle cerebral artery occlusion (pMCAO) using pericyte-deficient *Pdgfrb^+/–^* mice. Time-dependent reduction of infarct area sizes, i.e., repair, was significantly impaired in *Pdgfrb^+/–^* mice with recovery of cerebral blood flow (CBF) in ischemic areas attenuated by defective leptomeningeal arteriogenesis and intrainfarct angiogenesis. Peri-infarct astrogliosis, accompanied by increased STAT3 phosphorylation, was attenuated in *Pdgfrb^+/–^* mice. Pericyte-conditioned medium (PCM), particularly when treated with platelet-derived growth factor subunit B (PDGFB) homodimer (PDGF-BB; PCM/PDGF-BB), activated STAT3 and enhanced the proliferation and activity of cultured astrocytes. Although peri-infarct proliferation of oligodendrocyte (OL) precursor cells (OPCs) was induced promptly after pMCAO regardless of intrainfarct repair, OPC differentiation and remyelination were significantly attenuated in *Pdgfrb^+/–^* mice. Consistently, astrocyte-CM (ACM) promoted OPC differentiation and myelination, which were enhanced remarkably by adding PCM/PDGF-BB to the medium. Post-stroke functional recovery correlated well with the extent and process of intrainfarct repair and peri-infarct oligodendrogenesis. Overall, pericyte-mediated intrainfarct fibrotic repair through PDGFRβ may promote functional recovery through enhancement of peri-infarct oligodendrogenesis as well as astrogliosis after acute ischemic stroke.

## Visual Abstract

**Figure:**
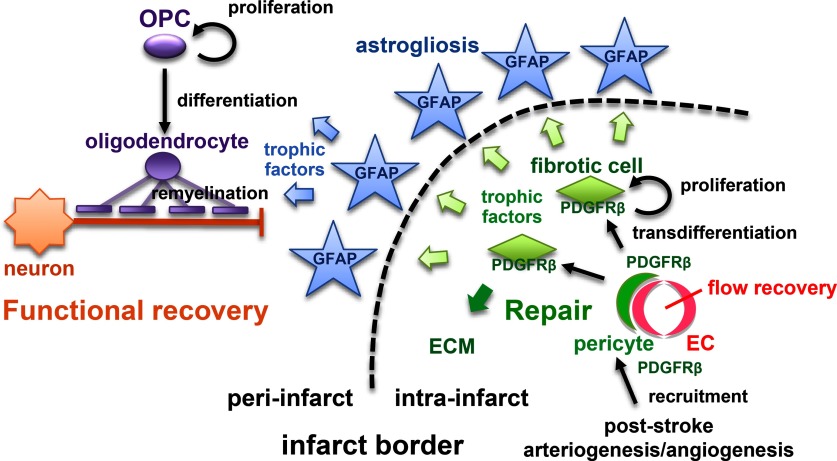

## Significance Statement

Pericyte-mediated fibrotic tissue repair is a major histological change within the infarct area during the subacute phase after ischemic stroke. Whether fibrotic repair is beneficial or detrimental to post-stroke functional recovery is highly debated. Here, we demonstrate that inhibition of fibrotic repair in mice by heterozygous deletion of platelet-derived growth factor receptor β (PDGFRβ) (*Pdgfrb^+/–^*) significantly attenuates functional recovery after ischemic stroke. Pericyte-derived PDGFRβ-positive cells within the infarct area produced trophic factors that activated astrocytes, thereby enhancing peri-infarct astrogliosis. Furthermore, astrocytes, conditioned with PDGF-BB-stimulated pericyte culture medium, promoted oligodendrocyte (OL) differentiation and a myelinating response. Peri-infarct oligodendrogenesis and re-myelination within areas of astrogliosis was significantly attenuated in *Pdgfrb^+/–^* mice. Pericyte-mediated tissue repair is beneficial for post-stroke functional recovery and is a potential therapeutic target.

## Introduction

Stroke is a leading cause of death and disability worldwide. Extensive efforts have been made to explore neuroprotective and neurorestorative therapies against acute ischemic stroke. However, most clinical trials targeting acute phase neuroprotection, based on promising results in animal experiments, have ended in failure, largely because of the limited therapeutic time window (Ginsberg, 2008; Xing et al., 2017). Exceptions include reperfusion therapies, either by intravenous infusion of recombinant tissue plasminogen activator (National Institute of Neurological D and Stroke rt-PA Stroke Study Group, 1995) or by catheter-mediated thrombectomy (Goyal et al., 2016). To achieve neuroprotection, focus should be paid to roles of non-neuronal components in the brain, such as vascular cells and glial cells, i.e., the concept of the neurovascular unit (Lo et al., 2003). We should also note that post-stroke functional recovery can occur spontaneously for up to three to six months with the aid of rehabilitation therapy (Cramer, 2008). The extent of recovery is often different between individuals, even when infarcts are of similar size and localization. Epidemiological studies also demonstrate that various factors, including age, sex, and status of life-style diseases, can affect post-stroke functional recovery. These findings encourage the exploration of therapeutic strategies that promote recovery of function, i.e., neurorestoration, even during the subacute phase after ischemic stroke (Chen et al., 2014; Tachibana et al., 2017; Xing et al., 2017).

Neurogenesis, astrogliosis, and oligodendrogenesis can occur in peri-infarct areas during the subacute phase. Although post-stroke neurogenesis has been extensively studied as a promising strategy for functional recovery, neural stem cells (NSCs) migrating from the subventricular zone (SVZ) into peri-infarct areas mostly differentiate into reactive astrocytes and contribute to astrogliosis, despite the NSCs having the potential to differentiate into neurons (Ohab et al., 2006; Faiz et al., 2015). It was believed that peri-infarct astrogliosis formed by migrating NSCs and resident astrocytes hindered axonal regrowth and impaired functional recovery; however, accumulating evidence demonstrates that astrogliosis may not simply isolate necrotic from healthy tissue to prevent the spread of damage, but that it may aid axonal regrowth and remyelination after ischemic stroke (Zamanian et al., 2012; Faiz et al., 2015; Anderson et al., 2016). In addition to astrogliosis, oligodendrogenesis is now recognized as a promising therapeutic target to promote functional recovery, not only in demyelinating disorders (Tognatta and Miller, 2016) but also in ischemic stroke (Iwai et al., 2010; Zhang et al., 2013; Itoh et al., 2015). Oligodendrocyte (OL) precursor cells (OPCs) make up 5% of the glial population and are diffusely distributed in the normal adult brain. Locally pre-existing OPCs, in addition to OPCs migrating from the SVZ, can differentiate into OLs to accomplish remyelination at sites of brain injury with demyelination, including the so-called penumbra (Levine et al., 2001; Itoh et al., 2015; Rosenzweig and Carmichael, 2015). Remyelination can restore neuronal functions and also prevent axonal degeneration leading to neuronal death; therefore, post-stroke enhancement of oligodendrogenesis is a promising strategy for functional recovery.

In addition to the peri-infarct neural reorganization, intrainfarct fibrosis is a prominent pathological change, in which pericytes play a pivotal role, during the subacute phase after ischemic stroke. Pericytes are vascular mural cells interacting directly with endothelial cells in microvessels at a high ratio in the CNS (Armulik et al., 2005). They regulate angiogenesis, stability of the blood-brain barrier (BBB) formed by endothelial cells, and cerebral blood flow (CBF) through the actions of platelet-derived growth factor receptor β (PDGFRβ; Daneman et al., 2010; Armulik et al., 2011; Sweeney et al., 2016). Furthermore, pericyte-derived PDGFRβ-expressing cells function as fibrosis-forming cells after ischemic stroke (Göritz et al., 2011; Makihara et al., 2015). Although a difficulty of pericyte research in the CNS is the lack of pericyte-specific markers, some reports have demonstrated that PDGFRβ can be used as a pericyte marker, particularly after ischemic stroke: the expression of PDGFRβ is induced remarkably and exclusively in pericytes within infarct areas (Winkler et al., 2010; Arimura et al., 2012). In these situations, it is still controversial whether pericyte-mediated fibrotic repair is beneficial or detrimental to peri-infarct neural reorganization and functional recovery (Su et al., 2008; Göritz et al., 2011; Makihara et al., 2015; Tachibana et al., 2017; Dias et al., 2018; Gautam and Yao, 2018). Thus, this is an important issue to be solved even from the standpoint of the development of novel therapeutic strategies that promote post-stroke functional recovery. In the present study, using heterozygous *Pdgfrb* knock-out (*Pdgfrb^+/–^*) mice, we aimed to elucidate how pericyte-mediated tissue repair within infarct areas affects peri-infarct neural reorganization and whether it can lead to better functional recovery after acute ischemic stroke.

## Materials and Methods

### Animals

Animal experiments were conducted according to the Guidelines for Proper Conduct of Animal Experiments by the National Science Council of Japan. The Animal Care and Use Review Committee of the Kyushu university approved our animal procedures. 129S *Pdgfrb^+/–^* mice were purchased from The Jackson Laboratory (https://www.jax.org/strain/007846). 129S *Pdgfrb^+/–^* mice were backcrossed with C57BL/6 ten times to produce C57BL/6 *Pdgfrb^+/–^* mice. Homozygous deletion of *Pdgfrb* (*Pdgfrb*^−/−^) is embryonic lethal (Soriano, 1994); therefore, we used *Pdgfrb^+/–^* mice to characterize the roles of PDGFRβ after ischemic stroke. We used mainly male mice (and female mice when indicated) aged 8–15 weeks and weighing 20–30 g. Mice were bred and housed two per cage in the authors’ animal facility at 21°C and 65% humidity with a regulated 12/12 h light/dark cycle and with free access to food and water. All experiments were reported according to the ARRIVE guidelines.

### Blood pressure (BP) and heart rate (HR) measurement

Mouse BP and HR were measured using the non-invasive tail-cuff BP system (BP-2000, Visitech Systems). Briefly, mice were trained with 10 consecutive preliminary measurements, followed by 25 study measurements that were averaged for each individual animal. The procedure was considered successful when 20 out of the 25 measurements were valid. Mice were conditioned to the BP monitoring procedure for three consecutive days before experiments. At day 0 before pMCAO, the baseline BP was measured. BP was measured again at days 1, 3, 7, and 14 after pMCAO.

### Mouse stroke model

Mice were randomly assigned to the animal surgeon and were anesthetized by the inhalation of 2% isoflurane in air and maintained under anesthesia with 1.5% isoflurane. Rectal temperatures were maintained at 35–37°C with a heat lamp. CBF before and during ischemia was measured at the ipsilateral parietal bone (2 mm posterior and 4 mm lateral to bregma) using a laser Doppler flowmeter (PeriFlux System 5000, PERIMED). Focal cerebral ischemia was induced by permanent occlusion of the right middle cerebral artery (pMCAO) using a laser-induced photochemical reaction as described previously (Makihara et al., 2015). Briefly, the right jugular vein was exposed and a catheter was inserted into the superior vena cava for intravenous administration of a solution. After the right distal MCA was carefully exposed, a diode-pumped solid-state laser was used to irradiate the distal MCA at a wavelength of 561 nm with 6 mW of emitted power. Upon laser irradiation, a photosensitizing Rose bengal dye solution (20 mg/kg, Wako #184-00272) was administrated intravenously for 90 s. After 4 min of irradiation, the laser beam was refocused on the MCA just proximal to the first position, followed by another 4 min of irradiation. Then, the right common carotid artery was ligated with a 6–0 silk suture.

### Measurement of CBF by laser speckle flowmetry

Relative CBF was determined by laser speckle flowmetry, which obtains high-resolution 2D imaging (OMEGAZONE OZ-2, OMEGAWAVE, INC.) and has a linear relationship with absolute CBF values (Ayata et al., 2004). Recordings were performed through the skull under anesthesia with 1.5% isoflurane. The majority of the periosteum, which adheres to the skull, was removed with fine-tip forceps. For each recording, the skull surface was wiped with saline-soaked gauze. The mean CBF of the ischemic core, located 2 mm posterior and 4 mm lateral to bregma, was measured in identically sized regions of interest (ROIs; 900 pixels). The absolute CBF value is theoretically vulnerable to many experimental conditions, such as magnification of the charge-coupled device camera and room light, so it may not always be reliable (Ayata et al., 2004). Therefore, we used the relative CBF of an ROI expressed as a percentage of the CBF value of the ipsilateral ROI against that of the corresponding ROI in the contralateral hemisphere.

### Latex perfusion method to assess leptomeningeal collaterals

In another set of mice, leptomeningeal collaterals were visualized 7 d after pMCAO. Under deep pentobarbital anesthesia, the right atrium of the heart was incised to allow for venous outflow. The left ventricle of the heart was cannulated, and 2-ml saline was injected. Immediately after the saline injection, 0.5-ml white latex compound (product no. 563; Chicago Latex Products Inc) mixed with 50 μl/ml carbon black (Bokusai; Fuekinori Kogyo Co, Ltd) was injected. The injection pressure was about 150 mmHg as reported previously (Komori et al., 2019). The brains were fixed with 4% paraformaldehyde (PFA) in PBS for 2 d. Photographs of the brain were taken at 50× magnification to measure the vessel diameter of the leptomeningeal anastomosis. The distal MCA was identified from its branch angle and distinguished from the distal ACA or posterior cerebral artery. The diameter of the leptomeningeal anastomosis was measured at the point of confluence between the distal MCA and the distal ACA. Independent persons not involved in the animal procedures performed the measurements.

### Assessment of BBB breakdown

Permeability of the BBB in infarct areas on day 28 was assessed by the leakage of Evans blue dye (Sigma-Aldrich #E2129). Briefly, the brain was collected 60 min after a 2% solution of Evans blue dye (4 ml/kg body weight) was injected intravenously via the tail vein. After mice were euthanized and transcardially perfused with ice-cold saline, whole brains were fixed with 4% PFA in PBS.

### Immunohistochemistry and immunofluorescence

Mice were killed at the indicated day after pMCAO by intraperitoneal administration of pentobarbital (150 mg/kg body weight) and transcardially perfused with 20-ml ice-cold saline followed by 20-ml 4% PFA in PBS at 4°C. Whole brains were fixed with 4% PFA in PBS for approximately 3 d. PFA-fixed 2-mm-thick coronal slices were embedded in paraffin and sectioned at 4 μm. The sections were then deparaffinized, rehydrated through a graded series of ethanol solutions, and washed in PBS (Tachibana et al., 2017). After blocking with a solution of 5% skimmed milk or PBS containing 10% normal goat serum for 30 min at room temperature, the sections were incubated with primary antibodies: anti-microtubule-associated protein 2 (MAP2; 1:1000; Sigma-Aldrich #M4403), anti-PDGFRβ [1:100; Cell Signaling Technology (CST) #3169], anti-CD13 (1:200; R&D Systems #AF2335), anti-CD31 (1:200; BD Biosciences #550274), anti-glial fibrillary acidic protein (GFAP; 1:200; CST #3670), anti-OLIG2 (1:200; R&D Systems #AF2418), anti-APC (1:100; Calbiochem #OP80), anti-ionized calcium-binding adapter molecule 1 (IBA1; 1:500; Abcam #ab5076), anti-myelin basic protein (MBP; 1:500; Millipore #MAB386), anti-SMI312 (1:500; BioLegend #837904), anti-doublecortin (DCX; 1:1000; Millipore #AB2253), anti-Fibrin (1:1000; DAKO #A0080), anti-NG2 (1:200; Millipore #AB5320), anti-interleukin-6 (IL6; 1:200; R&D Systems #AF206), or anti-pSTAT3 (1:1000; CST #9145) at 4°C overnight. After washing with PBS/Triton X-100, the sections were incubated with appropriate secondary antibodies conjugated to Alexa Fluor dyes (Thermo Fisher Scientific) or stained with 3,3′-diaminobenzidine (DAB) using an appropriate kit (Nichirei). For DAB staining, endogenous peroxidase was inactivated with 0.3% hydrogen peroxide for 30 min before blocking with skimmed milk solution. The sections were observed on a BIOREVO BZ-9000 microscope (Keyence). The same conditions were used to stain and observe all sections. The same color tone in all sections was converted to grayscale and measured at the same threshold with ImageJ software. Independent persons not involved in animal procedures performed the image analysis. MAP2-negative areas, infarct volumes, and tissue atrophy were calculated using six sections spaced 400 μm apart (from bregma +1.4 mm to bregma –0.6 mm). The actual infarct volumes with corrections for edema were calculated as the volume of the contralateral hemisphere minus the non-infarcted volume of the ipsilateral hemisphere. Tissue atrophy was calculated as the MAP2-positive volume of the contralateral hemisphere minus that of the ipsilateral hemisphere (Shi et al., 2017). CD13-, CD31-, and Fibrin-positive areas were obtained using three sections spaced 400 μm apart (from bregma +1.0 mm to bregma –0.2 mm). Intrainfarct CD13- or CD31- positive areas (%) = (CD13- or CD31- positive areas within infarct areas)/(infarct areas) × 100. OLIG2-, APC-, and IBA1-positive cells in the striatum were calculated using three sections spaced 400 μm apart (from bregma +1.0 mm to bregma –0.2 mm). To determine the density of positive cells, four randomly selected squares (400 × 400 μm) in the striatum were analyzed. We evaluated the MBP/SMI312 ratio in the striatum in an area 100 μm from the infarct area edge. The DCX-positive area in an area from the SVZ to the infarct was examined in five sections spaced 400 μm apart (from bregma +1.4 mm to bregma –0.2 mm). Peri-infarct GFAP-positive areas were calculated using three sections spaced 400 μm apart (from bregma +1.0 mm to bregma –0.2 mm). To determine the density of positive cells, four randomly selected squares (400 × 400 μm) were analyzed. The number of GFAP and pSTAT3 double-positive cells was evaluated from three serial sections spaced 400 μm apart (from bregma +1.0 mm to bregma –0.2 mm) in the peri-infarct area on day 28. Six to eight animals per group were analyzed.

### Cortical whole mount immunostaining

Mice were euthanized on day 7 by intraperitoneal administration of pentobarbital and transcardially perfused with 20-ml ice-cold saline and 20-ml 4% PFA. For immunostaining, the cortical surface of the brain was dissected (Okyere et al., 2018). Whole mounts were subsequently blocked in 5% skimmed milk for 30 min at room temperature, then incubated at 4°C overnight in primary antibodies: anti-α smooth muscle actin (SMA; 1:100; Abcam #ab7817), anti-CD31 (1:200; BD Biosciences #550274), and anti-F4/80 (1:100; Abcam #ab6640). After washing with PBS/Triton X-100, whole mounts were incubated with appropriate secondary antibodies conjugated to Alexa Fluor dyes (Thermo Fisher Scientific) for 30 min. Whole mounts were then washed and embedded in Vectashield DAPI mounting medium (Vector Laboratories #H1500) in a 35-mm glass dish, cover slipped, then imaged on an inverted confocal microscope (Nikon A1R).

### Quantitative polymerase chain reaction (PCR)

Total RNA was prepared from whole ischemic hemisphere, ischemic area, or cultured cells using TRIzol (Thermo Fisher Scientific #15596018). Reverse-transcription PCR (RT-PCR) and quantitative real-time PCR were performed as described previously (Makihara et al., 2015). Total RNA was reverse transcribed with a ReverTra Ace qPCR RT kit (Toyobo #FSQ-101). Using the reverse transcription product as a template, quantitative PCR was performed using a LightCycler (Roche). The mRNA copy numbers were standardized using 18s ribosomal RNA (rRNA) as an internal control. Primer sequences are presented in Table 1.

**Table 1.** Primers used for PCR

Target gene	Forward primer (5′-3′)	Reverse primer (5′-3′)
Mouse primer sequences
*Adgre1*	TCTGCAGTGTCAGCTCAGAA	GAAGTCTGGGAATGGGAGCT
*αSMA*	CTGACAGAGGCACCACTGAA	CATCTCCAGAGTCCAGCACA
*Bdnf*	GGTATCCAAAGGCCAACTGA	CTTATGAATCGCCAGCCAAT
*Ccl2/Mcp1*	CCAAATGAGATCAGAACCTACAACT	CTAGTTCACTGTCACACTGGTCACT
*Cd31*	TGCAGGAGTCCTTCTCCACT	ACGGTTTGATTCCACTTTGC
*Cldn5*	CTGGACCACAACATCGTGAC	GCCGGTCAAGGTAACAAAGA
*Ctnnb1*	AGGGTGCTATTCCACGACTA	CACCCTTCTACTATCTCCTCCAT
*Desmin*	GACATCCGGGCTCAGTATGA	CGCGCAATGTTGTCCTGATA
*Fgf2*	GGCTGCTGGCTTCTAAGTGT	CCGTTTTGGATCCGAGTTTA
*Fn1*	AATGGAAAAGGGGAATGGAC	CTCGGTTGTCCTTCTTGCTC
*Gfap*	AAGGTTGAATCGCTGGAGGA	AAGGTTGAATCGCTGGAGGA
*Igf1*	TGGATGCTCTTCAGTTCGTG	GTCTTGGGCATGTCAGTGTG
*Mag*	TGCTCACCAGCATCCTCACG	AGCAGCCTCCTCTCAGATCC
*Mbp*	TACCTGGCCACAGCAAGTAC	GTCACAATGTTCTTGAAG
*Ntf3*	GATCCAGGCGGATATCTTGA	AGCGTCTCTGTTGCCGTAGT
*Pdgfb*	GGCCACACACCTTCTCTGAT	GTGGAGGAGCAGACTGAAGG
*Pdgfrb*	CACCTTCTCCAGTGTGCTGA	GGAGTCCATAGGGAGGAAGC
*Plp*	GTATAGGCAGTCTCTGCGCTGAT	AAGTGGCAGCAATCATGAAGG
*Tnfa*	CGTCAGCCGATTTGCTATCT	CGGACTCCGCAAAGTCTAAG
*Zo1*	GGGCCATCTCAACTCCTGTA	AGAAGGGCTGACGGGTAAAT
*18s rRNA*	AAACGGCTACCACATCCAAG	CCTCCAATGGATCCTCGTTA
Human primer sequences
*Ccl2/Mcp1*	GATCTCAGTGCAGAGGCTCG	TGCTTGTCCAGGTGGTCCAT
*Il6*	GAACTCCTTCTCCACAAGCG	TTTTCTGCCAGTGCCTCTTT
*Tnfa*	CACTAAGAATTCAAACTGGGGC	GAGGAAGGCCTAAGGTCCAC
*Pdgfrb*	AGGTGGTTGCACATTTGTCCAG	TGTGCAGCTGTGTTCTTGAGAC
*18s rRNA*	AAACGGCTACCACATCCAAG	CCTCCAATGGATCCTCGTTA

### Immunoblot analysis

Immunoblot analysis using brain tissues was performed as described previously (Arimura et al., 2012). Membranes were incubated for 1 h with ECL Advance blocking reagent (GE Healthcare #RPN418) at room temperature, and probed overnight at 4°C with primary antibodies: anti-PDGFRβ (1:1000; CST #3169), anti-β-actin (1:3000; Sigma-Aldrich #A5441), anti-p-AKT (1:1000; CST #4060), anti-pan-AKT (1:1000; CST #4691), anti-p-ERK (1:1000; CST #4370), anti-ERK (1:1000; CST #4695), anti-p-STAT3 (1:1000; CST #9145), anti-STAT3 (1:1000; CST #4904), anti-GFAP (1:1000; CST #3670), and anti-NeuN (1:1000; Millipore #MAB377). Membranes were then washed and incubated with secondary antibodies (1:100,000; CST #7074 or #7076) for 45 min at room temperature.

### *In vivo* proliferation and differentiation assay

EdU (5-ethynyl-2′-deoxyuridine; Thermo Fisher Scientific #A10044) was used to label proliferating cells. Intraperitoneal EdU injections (50 mg/kg) were performed once daily for 7 d beginning on day 1 after pMCAO. Mice were killed on day 28 and perfused with 20-ml ice-cold saline followed by 20-ml 4% PFA. Brains were removed, and 2-mm coronal slices were postfixed for 1 h in 4% PFA, then cryoprotected in 30% sucrose overnight. Frozen coronal slices were embedded in optimum cutting temperature compound and sectioned at 4 μm. EdU staining was conducted using a Click-iTTM EdU imaging kit (Thermo Fisher Scientific #A10338). Briefly, sections were washed three times with PBS. After blocking with a solution of 3% bovine serum albumin (BSA) in PBS for 30 min at room temperature, sections were incubated with a reaction cocktail containing Click-iTTM reaction buffer, CuSO_4_, Alexa Fluor 594 Azide and reaction buffer additive for 30 min while protected from light. The sections were washed once with PBS and then stained with antibodies anti-GSTπ (1:100; MBL #312) and anti-NeuN (1:100; Millipore #MAB377) at 4°C overnight. Sections were washed in PBS/Triton X-100, incubated with the secondary antibody for 30 min, washed in PBS/Triton X-100, and then coverslipped with Vectashield DAPI mounting medium (Vector Laboratories). To assess the number of newly generated mature OLs (GSTπ+/EdU+ cells) and mature neurons in the penumbra (NeuN+/EdU+ cells) on day 28, three serial sections per animal spaced 400 μm apart (from bregma +1.0 mm to bregma –0.2 mm) were used. Eight animals per group were analyzed.

### Behavioral tests

Neurological tests were performed pre-pMCAO and 1, 3, 7, 14, 21, and 28 d after pMCAO. Sensorimotor deficits were assessed by the rotarod, modified neurological severity score (mNSS), and cylinder tests. The rotarod test was performed as described previously (Gertz et al., 2012). Briefly, mice were placed on a rotating drum accelerating from 4 to 40 rpm over 300 s. The latency to fall off the drum was recorded. Preoperative training was performed for 3 d with three trials per day, the last three trials serving as preoperative baseline. Postoperative tests were performed three times with an interval of 10 min between attempts. The mNSS was performed as described previously (Yukami et al., 2015). Neurological function was graded on a scale of 0 to 14 (normal score, 0; maximal deficit score, 14). The mNSS is a composite of motor, sensory, reflex, and balance tests. In the scores for severity of injury, 1 point is awarded for the inability to perform the test or for the lack of a tested reflex; thus, the higher the score, the more severe the injury. The cylinder test was performed as described previously (Tachibana et al., 2017). A mouse was placed in a clear acrylic cylinder 9 cm in diameter and 20 cm in height and videotaped during the test. Two mirrors were placed behind the cylinder to record forelimb movements when the mouse was turned away from the camera. Forelimb use during the first contact against the wall after rearing was recorded using previously described criteria in a blinded manner. However, if the right forelimb slipped following the simultaneous use of both forelimbs, a “both” and a “left” movement were recorded. Twenty movements were recorded. The final score = (left forelimb movement – right forelimb movement)/(left forelimb movement + right forelimb movement + both movements). In this test, normal animals would score, on average, 0. Eight animals per group were analyzed.

### Cell culture

Human brain vascular smooth muscle cells (SMCs) were purchased from ScienCell Research and Laboratories and cultured in SMC medium containing 2% fetal bovine serum (FBS) and SMC growth supplement. Human brain microvascular pericytes were purchased from ScienCell and cultured in pericyte medium containing 2% FBS and pericyte growth supplement. Poly-l-lysine (PLL)-coated dishes were prepared (IWAKI). SU16f (10 nM, R&D Systems #3304) was used as a selective PDGFRβ inhibitor. We obtained primary astrocytes and OPCs from ICR mice at postnatal day 1 (P1) using a previously described protocol (Matsuda et al., 2019) with some modifications. To obtain mixed glial cell cultures, cerebral cortices were carefully dissected after stripping away the meninges. The tissue was digested with papain (Sigma-Aldrich #3125) and triturated with 60 mg/ml DNase I (Wako #04726771). After centrifugation (200 g, 5 min), the cell pellet was resuspended in α-minimum essential medium (MEM), and the suspension was passed through a 40-μm cell strainer (BD Falcon #352340). After centrifugation (200 g, 5 min), the cell pellet was resuspended in DMEM containing 10% FBS. These mixed glial cells were plated in T75 tissue culture flasks. The medium was renewed every 2–3 d. Seven days after plating, microglia and OPCs were detached from astrocyte monolayer sheets by shaking at 200 rpm at 37°C for 15–18 h. Detached cells were collected and plated onto uncoated 100-mm culture dishes to eliminate contaminating astrocytes and microglia. After incubation for 30 min, the nonadherent OPCs were collected and plated onto PLL-coated dishes, then cultured with DMEM/F12 (Thermo Fisher Scientific #12400024) supplemented with insulin (25 mg/ml, Sigma-Aldrich #097-06474), apo-transferrin (100 mg/ml, Sigma-Aldrich #34401-55), progesterone (20 nM, Sigma-Aldrich #P0130), putrescine (60 mM, Sigma-Aldrich #P5780), sodium selenite (30 nM, Sigma-Aldrich #S5261), human PDGF-AA (10 ng/ml, Peprotech #100-13A), bFGF (20 ng/ml, Peprotech #100-18B), and EGF (20 ng/ml, Peprotech #100-15). Four to 5 d after plating, the OPCs were used for experiments. After the shake-off procedure for the isolation of microglia and OPCs, the cells were treated with AraC (5 mM, Sigma-Aldrich #C1768,) for 2 d to eliminate proliferating cells. Cultures were shaken once more, and then trypsin EDTA solution was added to the flask to obtain the remaining astrocytes, which were transferred to uncoated tissue culture dishes and maintained in DMEM containing 10% FBS. Two to 3 d after plating, the astrocytes were used for assays.

### CM preparation

To prepare CM for treating astrocytes, pericytes were incubated in pericyte medium without FBS and pericyte growth supplement for 48 h but containing PBS or PDGF-BB (10 nM, Wako #16424031). Then, CM was collected [referred to as pericyte-CM (PCM)/PBS and PCM/PDGF-BB, respectively]. Primary astrocytes were treated with PCM and DMEM without serum at a ratio of 1:1 for 24 h. Control medium was prepared with pericyte medium without cultured pericytes and DMEM at a ratio of 1:1.

### *In vitro* cell proliferation assay

We performed a cell proliferation assay using the CellTiter96 Aqueous One Solution Cell Proliferation Assay kit (Promega Corporation #G3580), containing the tetrazolium dye, MTS, according to the manufacturer’s instructions. Briefly, primary astrocytes were seeded onto 96-well plates at 1 x 10^4^ cells/well. The cells were incubated with DMEM containing 10% FBS for 24 h, then the medium was replaced with CM either with or without anti-IL6 antibody (R&D Systems #AF206; *n* = 6 per group). In the proliferation assay, 20 μl of the assay reagent was added to each well containing the cells in 100 μl of culture medium. The plate was then incubated for 2 h in a humidified, 5% CO_2_, atmosphere, and the absorbance was recorded at 490 nm using a microplate reader. Background absorbance was corrected by subtracting the absorbance of the reference at a wavelength of 650 nm. We also examined proliferation of cultured astrocytes by immunofluorescence with EdU staining. EdU was added to culture medium 1 h before fixation with 4% PFA in PBS. EdU- and GFAP-double-positive cells were counted in six ROIs (200 × 200 μm) in each dish (*n* = 6 dishes).

### Scratch assay

Confluent astrocyte monolayers were scratched once across the diameter of the glass coverslip using a sterile 200-μl pipette tip. We removed the culture medium immediately after wounding and replaced it with CM. Quantification of wound area and its closure after 72 h were analyzed using ImageJ software.

### *In vitro* OPC differentiation assay

To prepare CM for evaluating OPC differentiation, astrocytes were incubated in DMEM and control PCM, DMEM and PCM/PBS, or DMEM and PCM/PDGF-BB for 24 h. The CM [referred to as astrocyte-CM (ACM), A-PCM/PBS, or A-PCM/PDGF-BB, respectively] were then collected and centrifuged at 200 g for 5 min to remove cells and debris. Primary OPCs were treated with each CM and OPC differentiation medium (N2 medium with 50 ng/ml T3 and T4, Sigma-Aldrich #T2877 and #T2376) at a ratio of 1:2 for 5 or 7 d. Control CM was prepared from an empty dish and diluted with N2 medium in the same way. Medium was replaced every 2 d.

### Immunocytochemistry

Cells were fixed in 4% PFA and processed for immunostaining. Cells were blocked for 1 h at room temperature with blocking solution (5% FBS and 0.3% Triton X-100). To analyze marker expression profiles the following primary antibodies were used: anti-OLIG2 (1:200; R&D Systems #AF2418) and anti-MBP (1:500; Millipore #MAB386). Stained cells were visualized with a fluorescence microscope (BIOREVO BZ-9000). Cell counting was conducted in a blinded manner by randomly selecting six fields for each dish.

### Statistical analysis

Statistical analyses were performed with Student’s *t* test (for two-group comparisons) and one-way ANOVA, followed by a post hoc Bonferroni’s comparison test (for comparison of multiple groups) using Prism 8 (GraphPad Software). All data are presented as the mean ± SEM; *p* < 0.05 was considered significant. The sample sizes were similar to those reported in previous publications (Tachibana et al., 2017; Komori et al., 2019) and are indicated in the relevant figure legends. No statistical method was used to predetermine sample size.

## Results

### Reduction in the size of MAP2-negative infarct areas in the subacute phase is attenuated in *Pdgfrb^+/–^* mice

We subjected wild-type and *Pdgfrb^+/–^* mice to pMCAO. Immunoblot analysis demonstrated that the baseline expression level of PDGFRβ was lower in brain homogenates of *Pdgfrb^+/–^* mice by ∼80% (*p <* 0.001, unpaired *t* test; Extended Data Fig. 1-1). We confirmed by immunoblot analysis that PDGFRβ was abundant in cultured pericytes, but not in cultured astrocytes or cortical neurons (Extended Data Fig. 1-2). We also confirmed that systolic BP (SBP), diastolic BP (DBP) and HR were not different between wild-type and *Pdgfrb^+/–^* mice (*n* = 10, each group; Table 2). However, post-stroke body-weight loss was prolonged in *Pdgfrb^+/–^* mice, compared with wild-type mice (day 1, *p* = 0.317; day 3, *p* = 0.559; day 7, *p* = 0.055; day 14, *p* = 0.006; day 21, *p <* 0.001; day 28, *p <* 0.001, unpaired *t* test; Extended Data Fig. 1-3).

**Table 2.** Physiological data

		Base	1	3	7	14
Wild type	SBP	115 ± 7	98 ± 7	122 ± 10	122 ± 9	117 ± 9
*Pdgfrb^+/–^*	mmHg	114 ± 6	101 ± 13	117 ± 13	118 ± 12	117 ± 12
Wild type	DBP	62 ± 10	56 ± 11	67 ± 9	67 ± 7	65 ± 9
*Pdgfrb^+/–^*	mmHg	63 ± 5	58 ± 14	64 ± 14	61 ± 14	61 ± 7
Wild type	HR	551 ± 71	527 ± 106	558 ± 89	616 ± 68	616 ± 83
*Pdgfrb^+/–^*	bpm	572 ± 64	488 ± 76	556 ± 46	572 ± 55	643 ± 51

**Figure 1.**
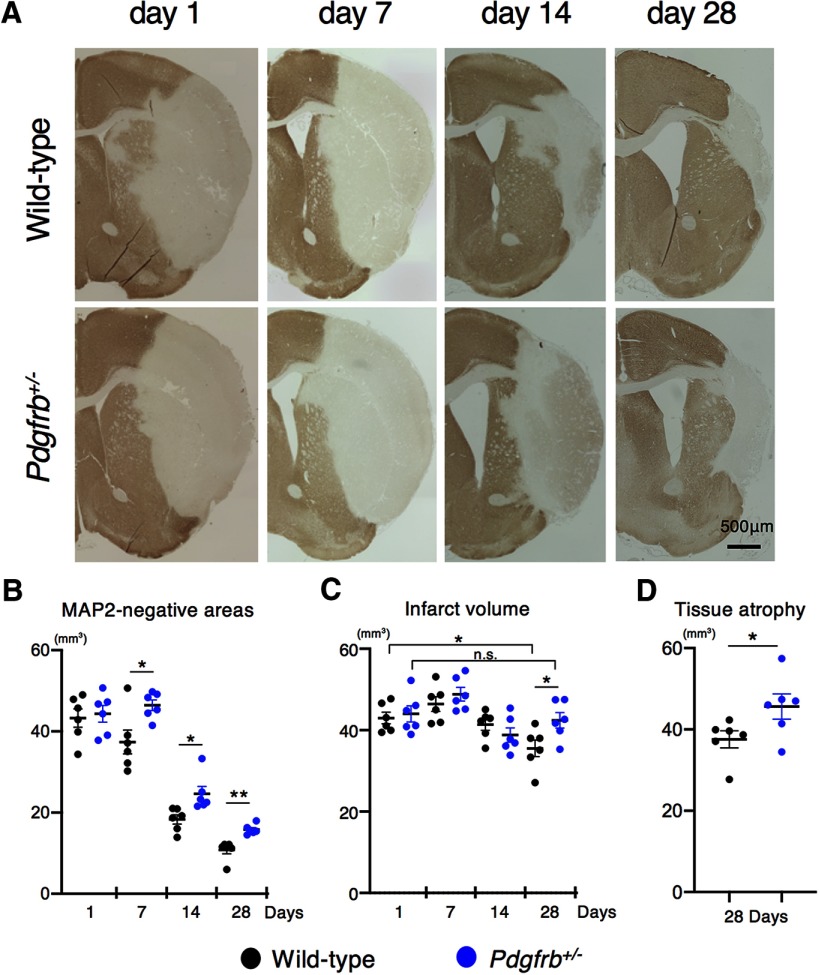
Reduction of MAP2-negative areas and infarct volume in the subacute phase is attenuated in *Pdgfrb^+/–^* mice. ***A***, MAP2 staining on days 1, 7, 14, and 28 after pMCAO in wild-type and *Pdgfrb^+/–^* mice (scale bar, 500 μm). ***B***, Quantification of MAP2-negative areas on days 1, 7, 14, and 28 after pMCAO (*n* = 6, each group). ***C,*** Quantification of infarct volume on days 1, 7, 14, and 28 after pMCAO (*n* = 6, each group). ***D,*** Quantification of tissue atrophy on day 28 after pMCAO (*n* = 6, each group). Data are shown as the mean ± SEM. ***B***, ***D***, *p < 0.05 and **p < 0.01, unpaired *t* test. ***C***, *p < 0.05, one-way ANOVA followed by Bonferroni’s *post hoc* test. n.s.: not significant.

10.1523/ENEURO.0474-19.2020.f1-1 Extended Data Figure 1-1 Baseline expression of PDGFRβ is decreased in the brain in *Pdgfrb^+/–^* mice. Immunoblot analysis demonstrates the baseline decrease in PDGFRβ expression by ∼80% in the brain in *Pdgfrb^+/–^* mice compared with wild-type mice (*n* = 4 mice per group). β-Actin is used as a loading control. Data represent the mean ± SEM (***p < 0.001, unpaired *t* test). Download Figure 1-1, DOCX file.

**Figure 2.**
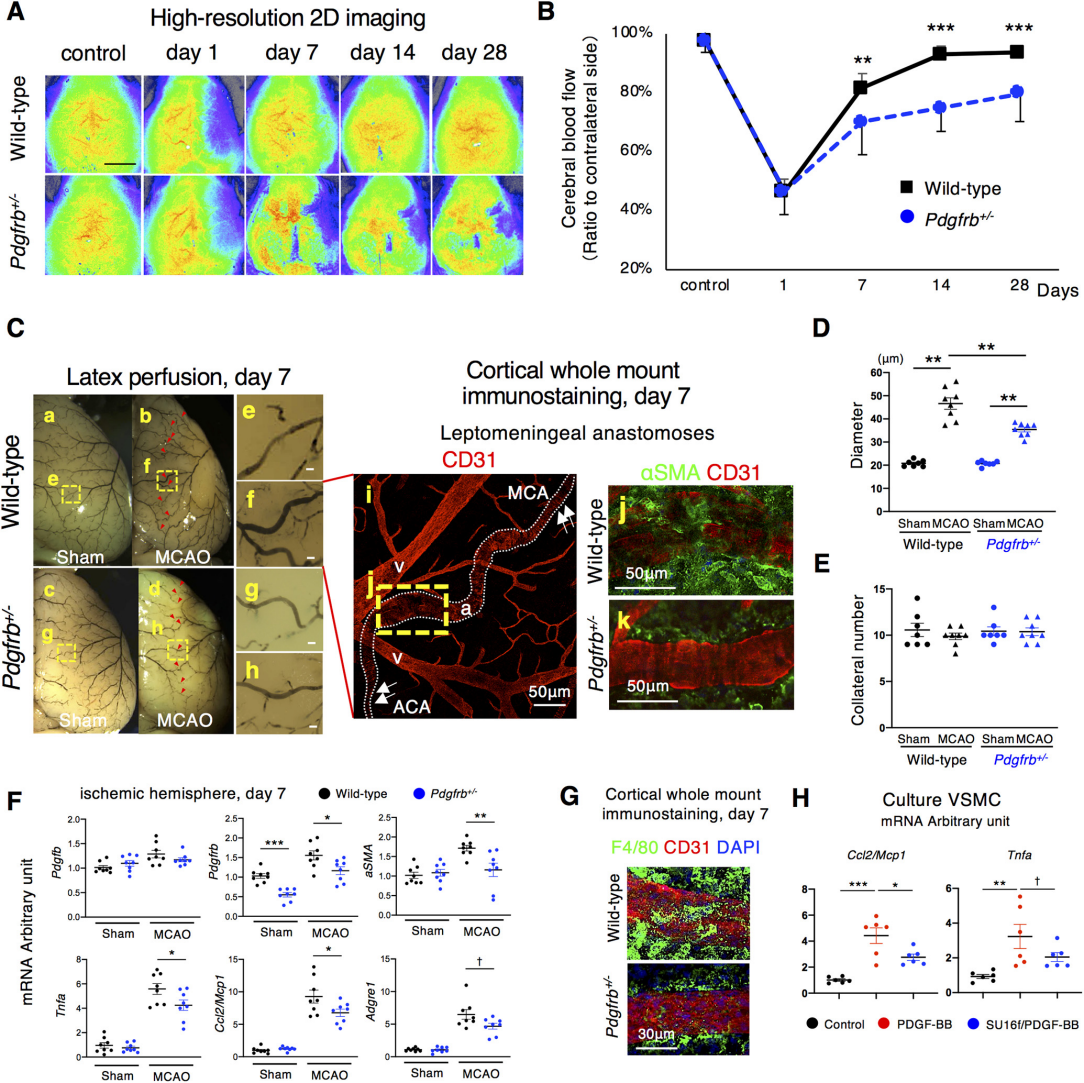
Leptomeningeal arteriogenesis and recovery of CBF in ischemic areas after pMCAO are attenuated in *Pdgfrb^+/–^* mice. ***A***, Images of CBF just under the cortical surface, as assessed by laser speckle flowmetry, in control and on days 1, 7, 14, and 28 after pMCAO in wild-type and *Pdgfrb^+/–^* mice (scale bar, 2 mm). ***B***, Temporal profiles of CBF quantification on days 1, 7, 14, and 28 after pMCAO (*n* = 12, each group). ***C***, Macroscopic images of leptomeningeal anastomoses, assessed by a latex perfusion method, at baseline (***a***, ***c***) and on day 7 (***b***, ***d***) after pMCAO in wild-type and *Pdgfrb^+/–^* mice (scale bar, 50 μm). Arrowheads indicate the leptomeningeal anastomoses vessels (***b***, ***d***). Magnified images of the dotted squares (***e****–****h***) in ***a****–****d*** are shown in the right panels. Cortical whole mount immunostaining of leptomeningeal anastomoses with CD31 (red) on day 7 after pMCAO is shown in ***i*** (a: artery, v: vein). Arrows indicate the pre-existing ACA and MCA. The magnified image of the yellow dotted square (***j***) is shown in the right panel. Double immunofluorescence labeling with CD31 (red) and αSMA (green) in anastomosed vessels on day 7 after pMCAO in wild-type (***j***) and *Pdgfrb^+/–^* mice (***k***) is shown (scale bar, 50 μm). ***D***, Diameter of anastomotic vessels at baseline and on day 7 after pMCAO (baseline, *n* = 7 each group; pMCAO, *n* = 8 each group). ***E***, Number of anastomotic vessels at baseline and on day 7 after pMCAO (baseline, *n* = 7 each group; pMCAO, *n* = 8 each group). ***F***, mRNA levels of arteriogenesis-related genes in non-infarct hemisphere and ischemic hemisphere on day 7 (*n* = 8, each group). ***G***, Cortical whole mount immunostaining with CD31 (red), F4/80 (green), and DAPI (blue) in wild-type and *Pdgfrb^+/–^* mice (scale bar, 50 μm). ***H***, Quantitative PCR for *Ccl2*/*Mcp1* and *Tnfa* in cultured VSMCs at baseline (black), with PDGF-BB (10 ng/ml; red), and with SU16f-pretreated PDGF-BB (100 nmol/l; blue; *n* = 6, each group). Data are shown as the mean ± SEM. ***B***, ***F***, ^†^*p* < 0.1, *p < 0.05, **p < 0.01, and ***p < 0.001, unpaired *t* test. ***D***–***E***, ***H***, ^†^*p* < 0.1, *p < 0.05, **p < 0.01, and ***p < 0.001, one-way ANOVA followed by Bonferroni’s *post hoc* test.

10.1523/ENEURO.0474-19.2020.f1-2 Extended Data Figure 1-2 Immunoblot analyses of PDGFRβ and cell-specific genes in cultured cells. Immunoblot analyses of PDGFRβ, GFAP, NeuN, and β-actin in cultured pericytes, astrocytes, and cortical neurons. Primary cortical neurons were isolated from ICR mice at embryonic day 17 (E17) as described previously (Nakashima et al., 2018).Download Figure 1-2, DOCX file.

**Figure 3.**
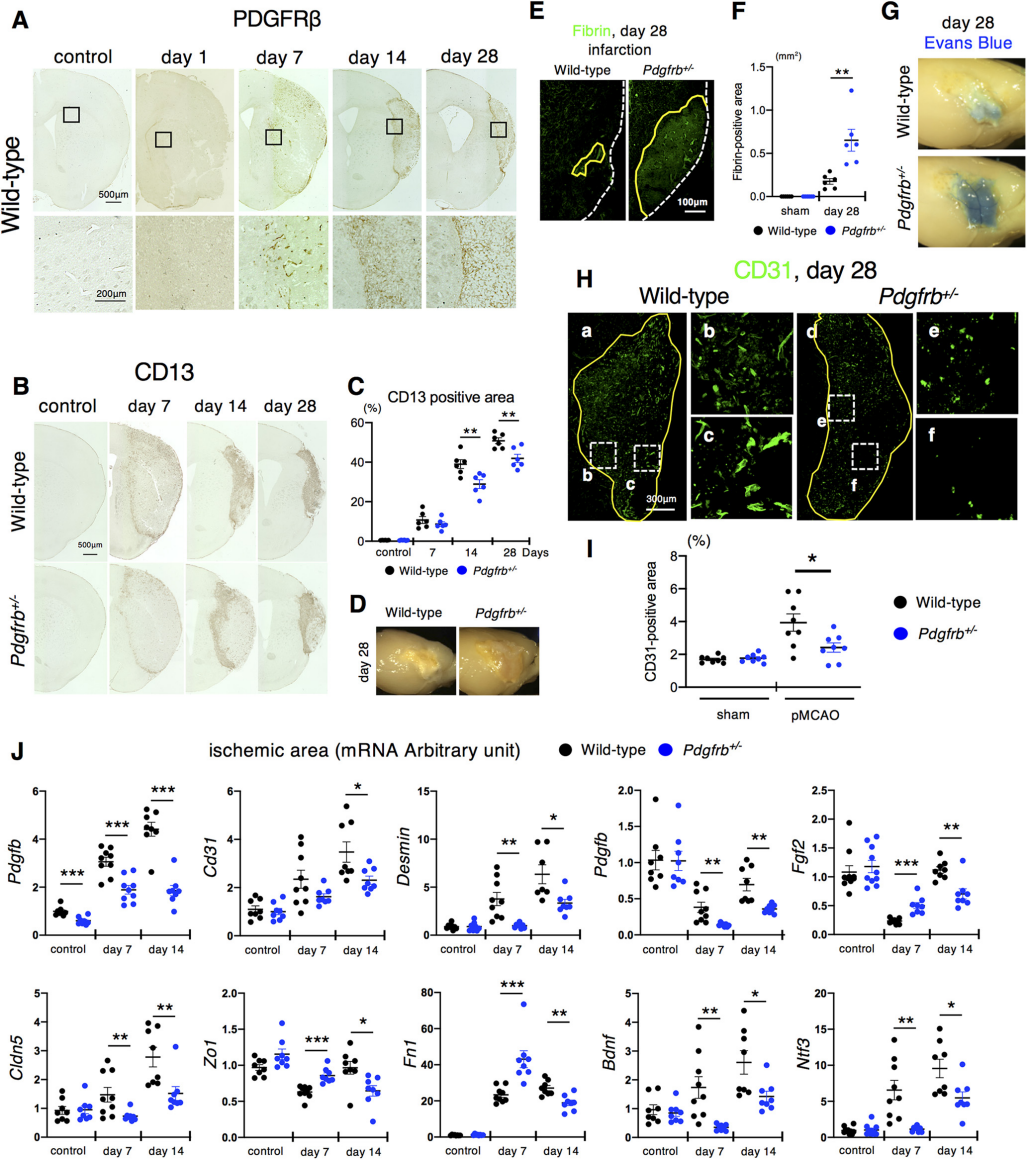
PDGFRβ is important for intrainfarct angiogenesis and fibrosis. ***A***, Immunohistochemistry of PDGFRβ at baseline and on days 1, 7, 14, and 28 after pMCAO (scale bar, 500 μm). Magnified images in the squares are shown at the bottom. ***B***, Immunohistochemistry of CD13, a marker of pericyte and pericyte-derived cells, in control and on days 7, 14, and 28 after pMCAO (scale bar, 500 μm). ***C***, Quantification of CD13-positive areas in control and on days 7, 14, and 28 after pMCAO in wild-type and *Pdgfrb^+/–^* mice (*n* = 6, each group). ***D***, Representative macroscopic observation of the brain surface on day 28 after pMCAO (*n* = 10). ***E***, Fibrin staining on day 28 after pMCAO (scale bar, 100 μm). ***F***, Quantification of fibrin-positive areas in mice on day 28 after pMCAO and in sham-operated mice (*n* = 6, each group). ***G***, Representative macroscopic images of Evans blue dye leakage in the brain on day 28 after pMCAO (*n* = 6). ***H***, Immunofluorescence labeling of CD31 on day 28 after pMCAO in wild-type (***a***) and *Pdgfrb^+/–^* mice (***d***; scale bar, 300 μm). Magnified images in the dotted squares (***b***, ***c***, ***e***, ***f***) are shown to the right. ***I***, Quantification of intrainfarct CD31 density in mice on day 28 after pMCAO and in sham-operated mice (*n* = 8, each group). ***J***, Quantitative PCR of genes expressed in non-infarct control and within infarct areas on days 7 and 14 after pMCAO in wild-type (black) and *Pdgfrb^+/–^* mice (blue; *n* = 8, each group). Data are shown as the mean ± SEM. ***C***, ***F***, ***I***, *p < 0.05, and **p < 0.01, unpaired *t* test. ***J***, *p < 0.05, **p < 0.01, and ***p < 0.001, one-way ANOVA followed by Bonferroni’s *post hoc* test.

10.1523/ENEURO.0474-19.2020.f1-3 Extended Data Figure 1-3 Body weight recovery after pMCAO is suppressed in *Pdgfrb^+/–^* mice. The graph shows body weight changes in wild-type and *Pdgfrb^+/–^* mice at the indicated days after pMCAO (*n* = 12, each group). Data represent the mean ± SEM (^†^*p* < 0.1, **p < 0.01 and ***p < 0.001, unpaired *t* test). Download Figure 1-3, DOCX file.

Immunohistochemically, MAP2-negative areas, including infarct and edema areas, were comparable between wild-type and *Pdgfrb^+/–^* mice on day 1 after pMCAO (*p* = 0.741, unpaired *t* test); however, they were significantly larger in *Pdgfrb^+/–^* mice compared with wild-type mice from day 7 to 28 (day 7, *p* = 0.019; day 14, *p* = 0.015; day 28, *p* = 0.001, unpaired *t* test; Fig. 1*A*,*B*). Similarly, infarct volumes were comparable between *Pdgfrb^+/–^* and wild-type mice on day 1; however, they were significantly larger in *Pdgfrb^+/–^* mice on day 28 (*p* = 0.033, ANOVA; Fig. 1*C*). These data suggested that brain edema was greater in *Pdgfrb^+/–^* mice on days 7 and 14. Furthermore, tissue atrophy was significantly larger in *Pdgfrb^+/–^* mice on day 28 (*p* = 0.023, unpaired *t* test; Fig. 1*D*). However, we should note that the difference of these parameters between the groups was small.

### Leptomeningeal arteriogenesis accompanied by monocyte/macrophage accumulation that leads to recovery of blood flow within ischemic areas is attenuated in *Pdgfrb^+/–^* mice

We next examined post-stroke recovery of CBF within ischemic areas. Although CBF in ischemic areas, as assessed by high-resolution 2D laser speckle flowmetry, was reduced equally in wild-type and *Pdgfrb^+/–^* mice (47 ± 7% vs 46 ± 6%, *p* = 0.942, unpaired *t* test) on day 1 after pMCAO, its recovery was significantly suppressed in *Pdgfrb^+/–^* mice compared with wild-type mice on day 7 (70 ± 11% vs 82 ± 4%, *p* = 0.005, unpaired *t* test), day 14 (75 ± 8% vs 93 ± 5%, *p <* 0.001, unpaired *t* test), and day 28 (80 ± 9% vs 94 ± 3%, *p <* 0.001, unpaired *t* test; *n* = 12; Fig. 2*A*,*B*).

We then examined the extent of leptomeningeal arteriogenesis after pMCAO. The number and the average diameter of leptomeningeal anastomoses between the distal MCA and the distal anterior cerebral artery (ACA) at baseline, as assessed by a latex perfusion method, were similar between wild-type (11 ± 2 vessels, 20.8 ± 4.1 μm; Fig. 2*Ca*,*e*) and *Pdgfrb^+/–^* mice (10 ± 1 vessels, 20.8 ± 3.7 μm; Fig. 2*Cc*,*g*; *p* = 0.872, *p* = 0.931, *n* = 7, ANOVA; Fig. 2*D*,*E*). On day 7 after pMCAO, the MCA territory was retrogradely perfused through enlarged leptomeningeal collaterals (Fig. 2*Cb*,*d*). The average diameter of collaterals was significantly greater in wild-type (46.6 ± 11.0 μm; Fig. 2*Cf*) compared with *Pdgfrb^+/–^* mice (35.4 ± 8.2 μm; Fig. 2*Ch*; *n* = 8, *p* = 0.008, ANOVA; Fig. 2*D*), while the number of collaterals was not significantly different between the groups (10 ± 1 vessels vs 10 ± 1 vessels, *n* = 8, *p* = 0.376, ANOVA; Fig. 2*E*). Cortical whole mount staining with CD31 confirmed that the diameter of anastomosed points was increased compared with the surrounding pre-existing ACA and MCA (arrows) on day 7 (Fig. 2*Ci*). Double staining with CD31 and αSMA showed that greater numbers of αSMA-positive SMCs were accumulated at the anastomosed points in wild-type mice compared with *Pdgfrb^+/–^* mice (Fig. 2*Cj*,*k*).

We then investigated mRNA expression of arteriogenesis-related molecules in the ischemic hemisphere. Post-stroke expression of *aSMA* and *Pdgfrb* was significantly lower (*aSMA*, *p* = 0.009; *Pdgfrb*, *p* = 0.022, unpaired *t* test), while that of platelet-derived growth factor subunit B (*Pdgfb*) was not altered (*p* = 0.212, unpaired *t* test), on day 7 in *Pdgfrb^+/–^* mice (Fig. 2*F*). Furthermore, mRNA levels of *Tnfa* and *Ccl2*/*Mcp1*, a representative pro-inflammatory cytokine and chemokine that recruit monocytes/macrophages, were significantly lower on day 7 in *Pdgfrb^+/–^* mice (*Tnfa*, *p* = 0.049; *Ccl2*/*Mcp1*, *p* = 0.048, unpaired *t* test; Fig. 2*F*). Consistently, immunofluorescence staining demonstrated that the recruitment of F4/80-positive monocytes/macrophages around anastomosed points was attenuated in *Pdgfrb^+/–^* mice, compared with wild-type mice (Fig. 2*G*).

We further demonstrated using cultured vascular SMCs that treatment with PDGFB homodimer (PDGF-BB) significantly increased the mRNA levels of *Ccl2*/*Mcp1* (*p <* 0.001, ANOVA) and *Tnfa* (*p* = 0.009, ANOVA), while pretreatment of the cells with SU16f, an inhibitor of PDGFRβ, abolished the effects of PDGF-BB (*Ccl2*/*Mcp1*: *p* = 0.029; *Tnfa*: *p* = 0.092, ANOVA; Fig. 2*H*). These findings indicated that SMCs, same lineage cells to pericytes, expressed PDGFRβ and produced molecules that recruit monocytes/macrophages, which can promote remodeling of collateral vessels leading to arteriogenesis (Scholz et al., 2000; Sugiyama et al., 2011).

### Intrainfarct angiogenesis and fibrosis are attenuated in *Pdgfrb^+/–^* mice

Consistent with the concept that reperfusion after ischemia promotes the repair process within infarct areas (Tachibana et al., 2017), intrainfarct fibrotic formation, as assessed by immunohistochemical staining with pericyte markers PDGFRβ and CD13 (Montagne et al., 2018), gradually expanded from day 7 to 28 and led to the shrinkage of infarct areas in wild-type mice (Figs. 1*A*, 3*A*,*B*). However, CD13-positive fibrotic formation was significantly attenuated in *Pdgfrb^+/–^* mice (control, *p* = 0.607; day 7, *p* = 0.337; day 14, *p* = 0.009; day 28, *p* = 0.007, unpaired *t* test; Fig. 3*B*,*C*). Macroscopic observation of the brain surface supported impaired scar formation in ischemic areas in *Pdgfrb^+/–^* mice on day 28 after pMCAO (Fig. 3*D*). The breakdown of the BBB within ischemic areas, as assessed by microscopic extravasation of fibrin (*p* = 0.005, unpaired *t* test; Fig. 3*E*,*F*) and macroscopic extravasation of Evans blue dye (Fig. 3*G*), was greater on day 28 in *Pdgfrb^+/–^* mice. We further demonstrated that the number of CD31-positive endothelial cells within infarct areas on day 28 was significantly smaller in *Pdgfrb^+/–^* mice (2.4 ± 0.8% vs 3.9 ± 1.4% in wild type, *p* = 0.024, unpaired *t* test; Fig. 3*H*,*I*), indicating that PDGFRβ also participated in intrainfarct angiogenesis leading to recovery of CBF after pMCAO.

To elucidate the significance of PDGFRβ-positive fibrotic lesions, we examined gene expression in non-infarct control and within ischemic areas on days 7 and 14 in wild-type and *Pdgfrb^+/–^* mice. The baseline expression of genes examined here, except *Pdgfrb*, was not significantly different between wild-type and *Pdgfrb^+/–^* mice (Fig. 3*J*). The expression of genes related to endothelial cells (*Cd31*), pericytes [*Des* (*Desmin*)], growth factors (*Pdgfb* and *Fgf2*), tight-junction related proteins [*Cldn5* (*Claudin 5*) and *Zo1*], and extracellular matrix proteins [*Fn1* (*fibronectin 1*)] was significantly decreased in *Pdgfrb^+/–^* mice on day 14 after pMCAO (Fig. 3*J*). Furthermore, the expression of *Bdnf* and *Ntf3*, representative neurotrophic factors, was significantly decreased in *Pdgfrb^+/–^* mice (Fig. 3*J*), indicating that PDGFRβ-mediated repair in ischemic areas may exert some positive effects on peri-infarct neural reorganization during the subacute phase after pMCAO.

### PDGFRβ-mediated repair enhances peri-infarct astrogliosis

**Figure 4.**
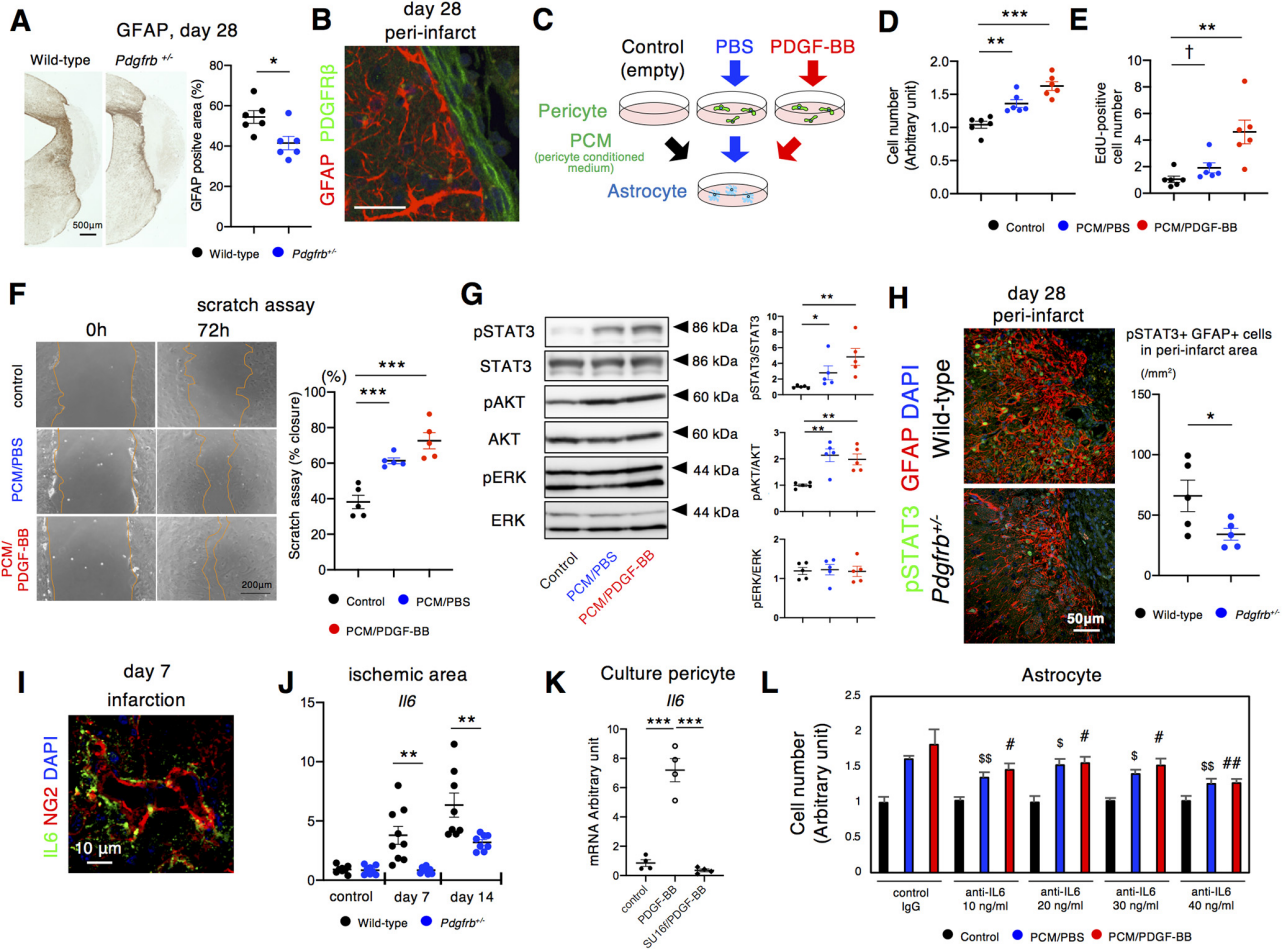
Pericyte-astrocyte interaction in peri-infarct areas after pMCAO. ***A***, Immunohistochemistry for GFAP (scale bar, 500 μm) and quantification of GFAP-positive areas on day 28 after pMCAO in wild-type and *Pdgfrb^+/–^* mice (*n* = 6, each group). ***B***, Double immunofluorescence labeling of GFAP (red) and PDGFRβ (green; scale bar, 20 μm) on day 28 after pMCAO. PDGFRβ-positive fibrotic lesion and GFAP-positive astrogliosis form a clear boundary. ***C***, Experimental scheme for phenotypic changes induced in astrocytes by pericyte culture medium (black, control), PCM treated with PBS (blue, PCM/PBS) or PDGF-BB (10 ng/ml; red, PCM/PDGF-BB). ***D***, MTT assay for astrocytes after treatment with PCM for 24 h (*n* = 6, each group). ***E***, Astrocyte proliferation assay as assessed by immunofluorescence of EdU after treatment with PCM for 24 h (*n* = 6, each group). ***F***, Astrocyte scratch assay after treatment with PCM for 72 h (scale bar, 200 μm; left). Quantification of wound recovery (*n* = 5, each group). ***G***, Immunoblot analyses of STAT3 and phospho-STAT3, AKT and phospho-AKT, and ERK and phospho-ERK in astrocytes after treatment with CM for 30 min (*n* = 5, each group). ***H***, Representative immunofluorescence labeling for pSTAT3 (green) and GFAP (red), and DAPI (blue) in the peri-infarct area after pMCAO in wild-type and *Pdgfrb^+/–^* mice on day 28 (scale bar, 50 μm). Quantification of the number of pSTAT3 and GFAP double-positive cells are evaluated (*n* = 5, each group). ***I***, Double immunofluorescence labeling of NG2 (red) and IL6 (green; scale bar, 10 μm) on day 7 after pMCAO. ***J***, Quantitative PCR of *Il6* expression in non-infarct hemisphere and within infarct areas on days 7 and 14 after pMCAO in wild-type (black) and *Pdgfrb^+/–^* mice (blue; *n* = 8, each group). ***K***, Expression change of *Il6* in pericytes treated with PDGF-BB in the presence or absence of SU16f (*n* = 4, each group). ***L***, MTT assay for astrocytes treated with PCM in the presence of anti-IL6 antibody (10–40 ng/ml; *n* = 6, each group). Data are shown as the mean ± SEM. ***A***, ***H***, ***J***, *p < 0.05, and **p < 0.01, unpaired *t* test. ***D***–***G***, ***K***, ***L***, ^†^*p* < 0.1, *p < 0.05, **p < 0.01, and ***p < 0.001; ^$^*p <* 0.05 and ^$$^*p <* 0.01 versus control IgG with PCM/PBS; and ^#^*p* < 0.05 and ^##^*p* < 0.01 versus control IgG with PCM/PDGF-BB, one-way ANOVA followed by Bonferroni’s *post hoc* test.

Consistent with previous studies (Shen et al., 2012; Makihara et al., 2015), we found that peri-infarct astrogliosis was significantly attenuated in *Pdgfrb^+/–^* mice (*p* = 0.018, unpaired *t* test; Fig. 4*A*) with a clear boundary to PDGFRβ-positive fibrotic areas (Fig. 4*B*). In contrast, the extent of IBA1-positive microglia/macrophage, localized in peri-infarct area, was not significantly different between wild-type and *Pdgfrb^+/–^* mice on day 28 after pMCAO (*p* = 0.465, unpaired *t* test; Extended Data Fig. 4-1*A*,*B*). Immunofluorescence staining demonstrated that IBA1-positive microglia/macrophage did not appear to express PDGFRβ (Extended Data Fig. 4-1*C*).

10.1523/ENEURO.0474-19.2020.f4-1 Extended Data Figure 4-1 Localization and extent of IBA1-positive microglia/macrophage on day 28 after pMCAO. ***A***, Representative IBA1 staining on day 28 after pMCAO in wild-type (left) and *Pdgfrb^+/–^* (right) mice. Scale bar, 500 μm. ***B***, Quantification of the number of IBA1-positive cells in peri-infarct areas on day 28 after pMCAO in wild-type (black) and *Pdgfrb^+/–^* mice (blue; *n* = 6, each group; *p* = 0.465, unpaired *t* test). ***C***, Representative immunofluorescence labeling for IBA1 (green) and PDGFRβ (red), and DAPI (blue) in peri-infarct area on day 28 after pMCAO in wild-type mice (Scale bar, 10 μm). Data represent the mean ± SEM. Download Figure 4-1, DOCX file.

To test a possible interaction between PDGFRβ-positive cells and GFAP-positive reactive astrocytes, we examined the effects of PCM treated with either PBS (PCM/PBS) or PDGF-BB (PCM/PDGF-BB) on cultured astrocytes (Fig. 4*C*). While PCM/PBS increased the number of astrocytes, PCM/PDGF-BB further enhanced the growth effect of PCM/PBS (PCM/PBS, *p* = 0.002; PCM/PDGF-BB, *p <* 0.001, ANOVA; Fig. 4*D*). A cell proliferation assay using EdU confirmed the growth effects of PCM/PBS and PCM/PDGF-BB on cultured astrocytes (PCM/PBS, *p* = 0.08; PCM/PDGF-BB, *p* = 0.003; ANOVA; Fig. 4*E*). Moreover, PCM/PBS enhanced astrocyte growth and migration after culture dishes were scratched, while PCM/PDGF-BB further enhanced the effect of PCM/PBS (PCM/PBS, *p <* 0.001; PCM/PDGF-BB, *p <* 0.001, ANOVA; Fig. 4*F*). Immunoblot analyses demonstrated that the phosphorylation of STAT3 and AKT, both crucial signaling molecules for the activation of astrocytes (Luo and Zheng, 2016), was significantly increased in astrocytes cultured with PCM/PBS (STAT3: *p* = 0.049, AKT: *p* = 0.002, ANOVA) and further increased with PCM/PDGF-BB (STAT3: *p* = 0.008; AKT: *p* = 0.002, ANOVA), while phosphorylation of ERK1/2 was not increased (Fig. 4*G*). Consistently, immunofluorescence staining demonstrated that STAT3 was strongly phosphorylated in GFAP-positive astrocytes in peri-infarct areas after pMCAO, which was attenuated in *Pdgfrb^+/–^* mice (*p* = 0.041, unpaired *t* test; Fig. 4*H*). It is known that IL6 cytokine family proteins activate STAT3 and AKT in astrocytes (Yamashita et al., 2005; Gertz et al., 2012); therefore, we examined whether IL6 was expressed in pericytes in ischemic areas *in vivo* (Nakamura et al., 2012). Double immunofluorescence labeling demonstrated that IL6 was expressed in NG2-positive pericytes (Fig. 4*I*), as well as in F4/80-positive macrophages and around CD31-positve endothelial cells (Extended Data Fig. 4-2), within the infarct area. Furthermore, the mRNA level of *Il6* was significantly increased in wild type on days 7 and 14 after pMCAO, compared with that of *Pdgfrb^+/–^* mice (day 7, *p* = 0.002; day 14, *p* = 0.009, unpaired *t* test; Fig. 4*J*). We confirmed that PDGF-BB increased the level of *Il6* mRNA in cultured pericytes (*p <* 0.001, ANOVA), while pretreatment with SU16f abolished the PDGF-BB-mediated upregulation of *Il6* (*p <* 0.001, ANOVA; Fig. 4*K*). Moreover, the growth effects of PCM on astrocytes were significantly attenuated by treatment with an anti-IL6 neutralizing antibody in a dose-dependent manner (Fig. 4*L*). These findings suggested the presence of intercellular interaction between PDGFRβ-positive pericytes and reactive astrocytes that is mediated, at least partly, through IL6.

10.1523/ENEURO.0474-19.2020.f4-2 Extended Data Figure 4-2 IL6 expression within infarct area after pMCAO. Double immunofluorescence labeling of and IL6 (green) and (***A***) F4/80 (red), a marker of macrophage (scale bar, 5 μm), or (***B***) CD31 (red), a marker of endothelial cell (scale bar, 10 μm), on day 7 after pMCAO. Download Figure 4-2, DOCX file.

### Peri-infarct oligodendrogenesis is attenuated in *Pdgfrb^+/–^* mice

**Figure 5.**
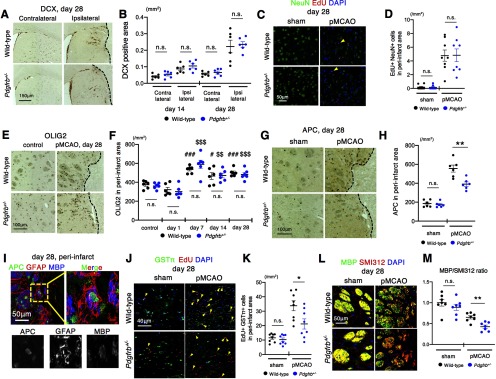
Peri-infarct oligodendrogenesis but not neurogenesis is attenuated in *Pdgfrb^+/–^* mice. ***A***, Immunohistochemistry for DCX in the SVZ and striatum on day 28 after pMCAO in wild-type and *Pdgfrb^+/–^* mice (scale bar, 150 μm). ***B***, Quantification of the DCX-positive areas in the ipsilateral and contralateral hemisphere on days 14 and 28 after pMCAO in wild-type (black) and *Pdgfrb^+/–^* mice (blue; *n* = 6, each group). ***C***, Double immunofluorescence labeling of NeuN (green) and EdU (red) in the peri-infarct areas on day 28 after pMCAO and in sham-operated mice. Arrowheads indicate NeuN and EdU double-positive newborn mature neurons (scale bar, 50 μm). ***D***, Quantification of the number of double-positive cells in peri-infarct areas on day 28 after pMCAO and in sham-operated mice (*n* = 8, each group). ***E***, Immunohistochemistry for OLIG2 in sham-operated mice and in peri-infarct areas on day 28 after pMCAO in wild-type and *Pdgfrb^+/–^* mice (scale bar, 100 μm). ***F***, Quantification of the number of OLIG2-positive cells in peri-infarct areas on days 1, 7, 14, and 28 after pMCAO in wild-type (black) and *Pdgfrb^+/–^* mice (blue; *n* = 6, each group). ***G***, Immunohistochemistry for APC in sham-operated mice and in peri-infarct areas on day 28 after pMCAO (scale bar, 100 μm). ***H***, Quantification of the number of APC-positive cells in peri-infarct areas on day 28 after pMCAO (*n* = 6, each group). ***I***, Triple immunofluorescence labeling with APC (green), GFAP (red) and MBP (blue) in peri-infarct areas (striatum) on day 28 after pMCAO. Magnified images of the dotted square are shown below and in the right panel (scale bar, 50 μm). ***J***, Double immunofluorescence labeling of GSTπ (green) and EdU (red) in sham-operated mice and in the peri-infarct areas on day 28 after pMCAO. Arrowheads indicate GSTπ and EdU double-positive newborn mature OLs (scale bar, 40 μm). ***K***, Quantification of the number of double-positive cells in peri-infarct areas on day 28 after pMCAO (*n* = 8, each group). ***L***, Double immunofluorescence labeling of MBP (green) and the pan-axonal neurofilament marker, SMI312 (red), in sham-operated mice and in peri-infarct striatum on day 28 after pMCAO (scale bar, 50 μm). ***M***, Quantification of MBP/SMI312 ratio in the peri-infarct striatum on day 28 after pMCAO (*n* = 7, each group). Data are shown as the mean ± SEM. ***B***, ***D***, ***H***, ***K***, ***M***, *p < 0.05 and **p < 0.01, unpaired *t* test. ***F***, ^#^*p* < 0.05 and ^###^*p* < 0.001 vs. control wild-type mice, ^$$^*p <* 0.01 and ^$$$^*p <* 0.001 vs. control *Pdgfrb^+/–^* mice, one-way ANOVA followed by Bonferroni’s post-hoc test. n.s.: not significant.

We further examined whether PDGFRβ-mediated repair could elicit neural reorganization other than astrogliosis in peri-infarct areas. We first examined the recruitment of DCX-positive immature neurons from the SVZ. Immunohistochemistry for DCX demonstrated that the recruitment of the immature neurons in the ipsilateral hemisphere was not significantly different between wild-type and *Pdgfrb^+/–^* mice on days 14 and 28 after pMCAO (day 14, *p* = 0.302; day 28, *p* = 0.766, unpaired *t* test; Fig. 5*A*,*B*). Furthermore, the number of EdU- and NeuN-double positive newly generated neurons in peri-infarct areas was not different on day 28 between the groups (5 ± 2 vs 5 ± 3 cells/mm^2^, *p* = 0.886; unpaired *t* test; Fig. 5*C*,*D*). We then examined oligodendrogenesis in peri-infarct areas. The number of OLIG2-positive OPCs was significantly increased from day 7 to 28 equally in wild-type and *Pdgfrb^+/–^* mice (day 7, *p* = 0.272; day 14, *p* = 0.897; day 28, *p* = 0.489, unpaired *t* test; Fig. 5*E*,*F*). However, we found that the number of APC-positive differentiated OLs in peri-infarct areas was significantly greater in wild-type compared with *Pdgfrb^+/–^* mice on day 28 (555 ± 83 vs 391 ± 52 cells/mm^2^, *p* = 0.004, unpaired *t* test; Fig. 5*G*,*H*). Furthermore, triple immunofluorescence staining demonstrated that the oligodendrogenesis and myelination, as shown by APC (green) and MBP (blue) staining, occurred within GFAP (red)-positive astrogliosis in peri-infarct areas (Fig. 5*I*). Double immunofluorescence labeling with EdU and GSTπ demonstrated that the number of newly generated OLs in peri-infarct areas was significantly greater on day 28 in wild-type than in *Pdgfrb^+/–^* mice (34 ± 9 vs 21 ± 9 cells/mm^2^, *p* = 0.022, unpaired *t* test; Fig. 5*J*,*K*). Moreover, the extent of axon myelination, as assessed by double immunofluorescence labeling with MBP and the pan-axonal neurofilament marker, SMI312, was significantly greater on day 28 in wild-type mice than in *Pdgfrb^+/–^* mice (*p* = 0.002, unpaired *t* test; Fig. 5*L*,*M*). We confirmed that the extent of demyelination in the striatum and the corpus callosum on day 1 after pMCAO was not different between the groups (striatum, *p* = 0.394; corpus callosum, *p* = 0.920, unpaired *t* test; Extended Data Fig. 5-1), indicating that post-stroke peri-infarct remyelination was significantly attenuated in *Pdgfrb^+/–^* mice.

10.1523/ENEURO.0474-19.2020.f5-1 Extended Data Figure 5-1 White matter injury after pMCAO is comparable between wild-type and *Pdgfrb^+/–^* mice. ***A***, ***B***, Representative Klüver–Barrera (KB) staining on day 1 after pMCAO in wild-type and *Pdgfrb^+/–^* mice (scale bar, 500 μm). ***C***, ***D***, Magnified images of peri-infarct striatum are shown (scale bar, 100 μm). ***E***, ***F***, The extent of white matter injury is assessed by KB-positive area in striatum and corpus callosum against that of the contralateral hemisphere (*n* = 6, each group, unpaired *t* test). Data represent the mean ± SEM. Download Figure 5-1, DOCX file.

### Astrocytes that are stimulated with PCM produce factors promoting differentiation and myelination of OPCs

**Figure 6.**
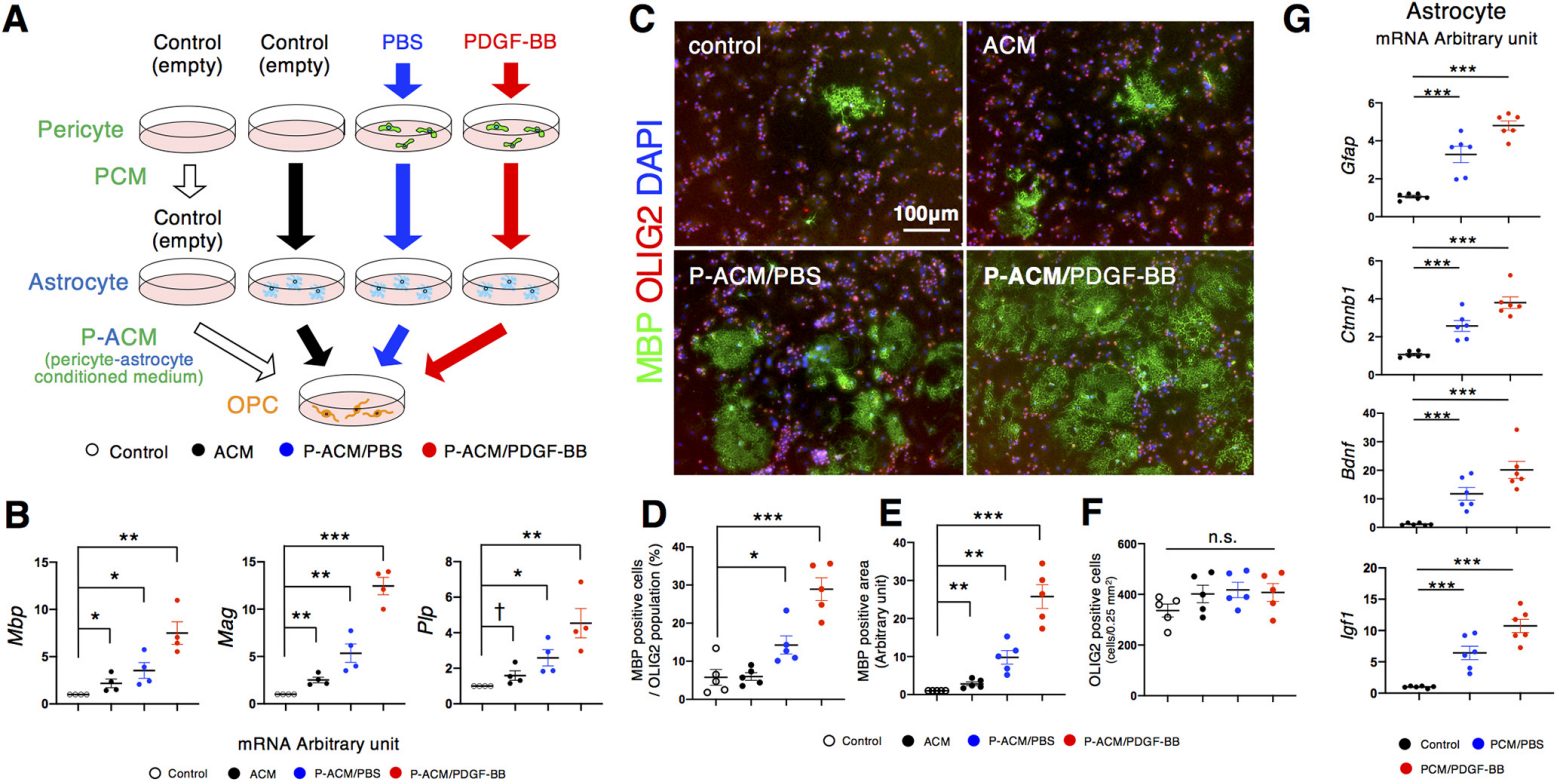
Astrocytes stimulated with PCM promote differentiation and myelination. ***A***, An experimental scheme for OPC differentiation in normal culture medium (control) or in astrocyte culture CM pretreated with empty pericyte medium, PCM/PBS, or PCM/PDGF-BB. ***B***, Quantitative PCR of *Mbp*, *Mag*, and *Plp* in OPCs stimulated with CM for 5 d (*n* = 4, each group). ***C***, Double immunofluorescence labeling of MBP (green) and OLIG2 (red) in cultured OPCs treated with ACM for 7 d (*n* = 5, each group; scale bar, 100 μm). ***D–F***, Quantification of the number of MBP-positive cells among OLIG2-positive cells (***D***), MBP-positive areas (***E***), and OLIG2-positive cells (***F***). ***G***, Quantitative PCR for *Gfap*, *Ctnnb1*, *Bdnf*, and *Igf1* in astrocytes treated with PCM for 24 h (*n* = 6, each group). Data are shown as the mean ± SEM. ***B***, ***D–G***, ^†^*p* < 0.1, *p < 0.05, **p < 0.01, and ***p < 0.001, one-way ANOVA followed by Bonferroni’s *post hoc* test. n.s.: not significant.

To elucidate how the differentiation of OPCs was induced in peri-infarct areas, we examined the intercellular interaction among pericytes, astrocytes, and OPCs, using cultured cells. We tested whether ACM, ACM treated with PCM/PBS (P-ACM/PBS), or ACM treated with PCM/PDGF-BB (P-ACM/PDGF-BB) could promote the proliferation, differentiation and myelination of cultured OPCs (Fig. 6*A*). We examined expression of *Mbp*, myelin associated glycoprotein (*Mag*), and myelin proteolipid protein (*Plp*), markers of myelination, in OPCs by quantitative PCR. The largest increase in expression of these markers was seen after treatment with P-ACM/PDGF-BB, followed by P-ACM/PBS and then ACM (*Mbp*: ACM, *p* = 0.044; P-ACM/PBS, *p* = 0.026; P-ACM/PDGF-BB, *p* = 0.002; *Mag*: ACM, *p* = 0.002; P-ACM/PBS, *p* = 0.004; P-ACM/PDGF-BB, *p <* 0.001; *Plp*: ACM, *p* = 0.069; P-ACM/PBS, *p* = 0.013; P-ACM/PDGF-BB, *p* = 0.005, ANOVA; Fig. 6*B*). Consistently, double immunofluorescence labeling showed that the number of MBP-positive cells among OLIG2-positive cells (ACM, *p* = 0.930; P-ACM/PBS, *p* = 0.028; P-ACM/PDGF-BB, *p <* 0.001, ANOVA; Fig. 6*C*,*D*) and in MBP-positive areas (ACM, *p* = 0.007; P-ACM/PBS, *p* = 0.001; P-ACM/PDGF-BB, *p <* 0.001, ANOVA; Fig. 6*C*,*E*) were greatest in OPCs treated with P-ACM/PDGF-BB, while the number of OLIG2-positive cells was not significantly different among the groups (Fig. 6*C*,*F*). We also examined the direct effects of PCM on OPCs. Although the numbers of MBP-positive cells among OLIG2-positive ones and MBP-positive areas were greater in OPCs treated with PCM/PBS and PCM/PDGF-BB, their extents were much lower compared with those treated with ACM (Fig. 6; Extended Data Fig. 6-1). These findings indicated that astrocytes, stimulated with pericyte-derived molecules, may promote the differentiation and myelination of near-by OPCs.

10.1523/ENEURO.0474-19.2020.f6-1 Extended Data Figure 6-1 Effects of PCM on OPC differentiation and myelination. ***A***, Double immunofluorescence labeling of MBP (green) and OLIG2 (red) in cultured OPC treated with PCM for 7 d (*n* = 5, each group; scale bar, 100 μm). ***B***, An experimental scheme for OPC differentiation in normal culture medium (control) or in PCM treated with PBS (blue, PCM/PBS) or PDGF-BB (10 ng/ml; red, PCM/PDGF-BB). ***C***, ***D***, Quantification of the number of MBP-positive cells within OLIG2-positive OPCs and MBP-positive areas. Data represent the mean ± SEM (*p < 0.05, **p < 0.01, and ***p < 0.001 one-way ANOVA followed by Bonferroni’s *post hoc* test). Download Figure 6-1, DOCX file.

We confirmed by quantitative PCR that PCM, particularly when conditioned with PDGF-BB, increased the expression of *Gfap* and *Ctnnb1* (β-catenin), markers of reactive astrocytes (Cregg et al., 2014; Hara et al., 2017), and of *Bdnf* and *Igf1*, trophic factors that promote OPC differentiation and myelination, in cultured astrocytes (*Gfap*: PCM/PBS, *p <* 0.001; PCM/PDGF-BB, *p <* 0.001: *Ctnnb1*: PCM/PBS, *p <* 0.001; PCM/PDGF-BB, *p <* 0.001: *Bdnf*: PCM/PBS, *p <* 0.001; PCM/PDGF-BB, *p <* 0.001: *Igf1*: PCM/PBS, *p <* 0.001; PCM/PDGF-BB, *p <* 0.001, ANOVA; Fig. 6*G*).

### Functional recovery after pMCAO is significantly attenuated in *Pdgfrb^+/–^* mice

**Figure 7.**
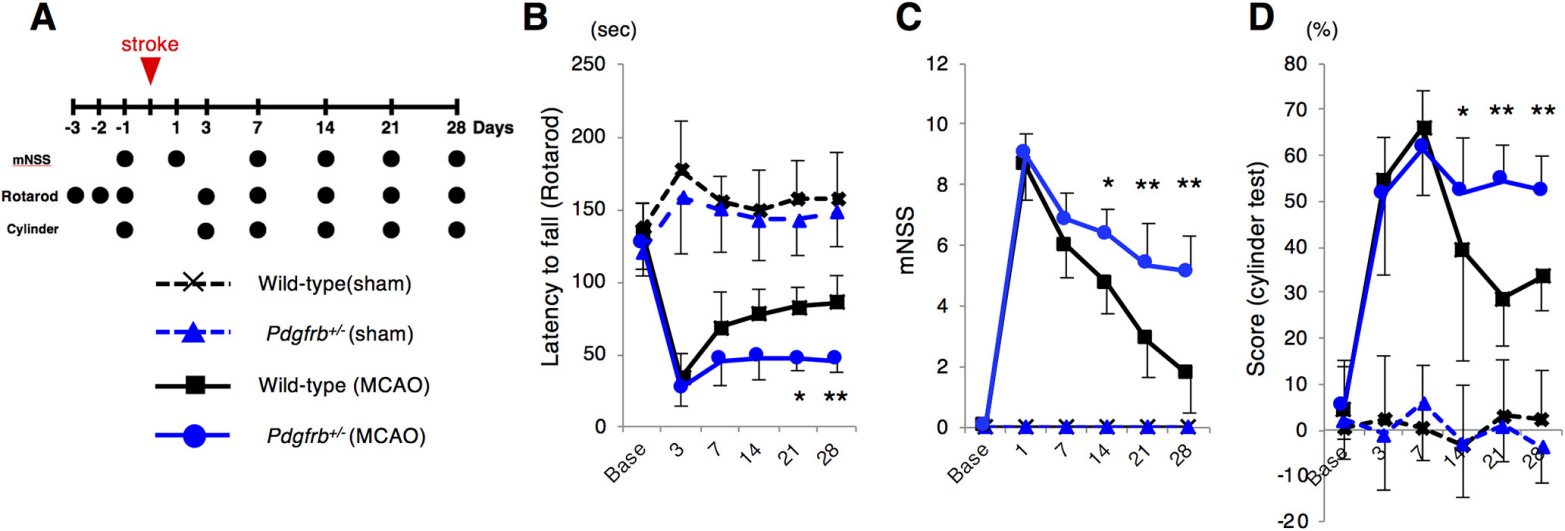
Functional recovery after pMCAO was attenuated in *Pdgfrb^+/–^* mice. ***A***, Experimental schedule for neurological assessment. Neurological function was assessed by (***B***) rotarod test, (***C***) mNSS test, and (***D***) cylinder test at baseline and days 1, 3, 7, 14, 21, and 28 after pMCAO in wild-type (black) and *Pdgfrb^+/–^* mice (blue). Data are shown as the mean ± SEM (*n* = 8, each group, *p < 0.05, and **p < 0.01, unpaired *t* test).

We finally tested whether the peri-infarct neural reorganization, including astrogliosis and oligodendrogenesis, enhanced by PDGFRβ-mediated repair was associated with functional recovery after pMCAO, using wild-type and *Pdgfrb^+/–^* mice (Fig. 7*A*). Neurological functions, assessed by rotarod, mNSS, and cylinder tests, were decreased similarly in both mice on days 1–3; however, functional recovery after day 14 was significantly better in wild-type mice than in *Pdgfrb^+/–^* mice, consistent with the temporal profiles of intrainfarct repair and peri-infarct neural reorganization (Fig. 7*B–D*).

### Post-stroke phenotypic changes in female *Pdgfrb^+/–^* mice

**Figure 8.**
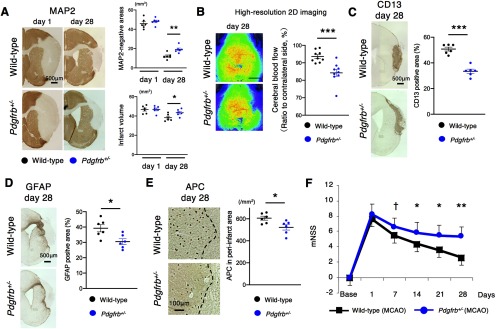
Post-stroke phenotypic changes in female *Pdgfrb^+/–^* mice. ***A***, MAP2 staining on days 1 and 28 after pMCAO in female wild-type and *Pdgfrb^+/–^* mice (scale bar, 500 μm). Quantification of MAP2-negative areas and infarct volume (*n* = 6, each group). Data are shown as the mean ± SEM (*p < 0.05 and **p < 0.01, unpaired *t* test). ***B***, Representative laser speckle images of CBF after pMCAO in female wild-type and *Pdgfrb^+/–^* mice on day 28 (*n* = 9, each group). Scale bar, 2 mm. Data represent the mean ± SEM (***p < 0.001, unpaired *t* test). ***C***, Representative CD13 staining (scale bar, 500 μm) and quantification on day 28 after pMCAO in female wild-type and *Pdgfrb^+/–^* mice (*n* = 6, each group). Data are shown as the mean ± SEM (***p < 0.001, unpaired *t* test). ***D***, Representative GFAP staining (scale bar, 500 μm) and quantification on day 28 after pMCAO in wild-type and *Pdgfrb^+/–^* female mice (*n* = 6, each group). Data are shown as the mean ± SEM (*p < 0.05, unpaired *t* test). ***E***, Representative APC staining (scale bar, 100 μm) and quantification on day 28 after pMCAO in female wild-type and *Pdgfrb^+/–^* mice (*n* = 6, each group). Data are shown as the mean ± SEM (*p < 0.05, unpaired *t* test). ***F***, Neurological scores in female mice after pMCAO (*n* = 9, each group). Data represent the mean ± SEM (^†^*p* < 0.1, *p < 0.05, and **p < 0.01, unpaired *t* test).

We also tested post-stroke phenotypic changes in female *Pdgfrb^+/–^* mice. The reduction of MAP2-negative areas and infarct volume was significantly attenuated (MAP2-negative areas, *p* = 0.004; infarct volume, *p* = 0.029, unpaired *t* test; Fig. 8*A*) with the recovery of CBF attenuated in ischemic areas (*p <* 0.001, unpaired *t* test; Fig. 8*B*) on day 28 after pMCAO. CD13-positive fibrotic formation was significantly attenuated (*p <* 0.001, unpaired *t* test; Fig. 8*C*) accompanied with reduced GFAP-positive astrogliosis (*p* = 0.016, unpaired *t* test; Fig. 8*D*) and APC-positive differentiated OLs (*p* = 0.023, unpaired *t* test; Fig. 8*E*) in peri-infarct areas on day 28. Consistently, post-stroke functional recovery was significantly impaired in female *Pdgfrb^+/–^* mice (Fig. 8*F*).

## Discussion

**Figure 9.**
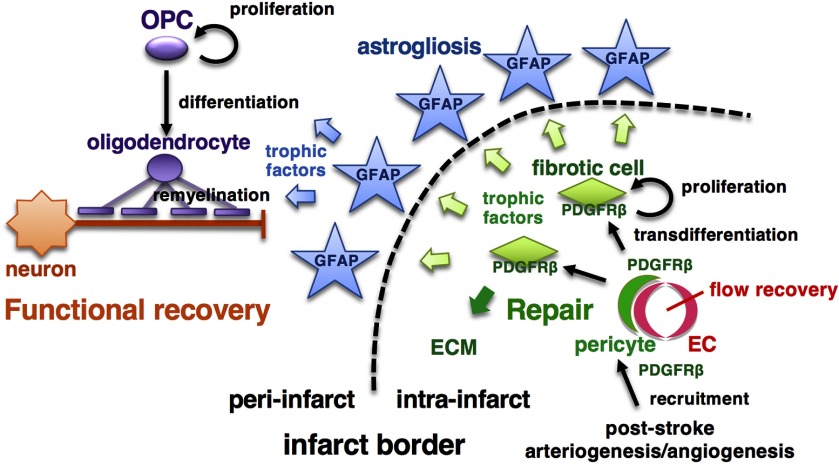
Schematic diagram of pericyte-mediated tissue repair and functional recovery after pMCAO. Schematic diagram of this study. PDGFRβ signaling in vascular mural cells plays important roles in mediating post-stroke leptomeningeal arteriogenesis and intrainfarct angiogenesis leading to the recovery of CBF in ischemic areas. After recruitment around endothelial cell (EC) tubes, PDGFRβ-positive pericytes transdifferentiate into fibrotic cells, enhance tissue repair within infarct areas and astrogliosis in peri-infarct areas, and promote peri-infarct oligodendrogenesis (which together is OPC differentiation and myelination) leading to functional recovery. ECM, extracellular matrix.

We extensively examined the roles of pericytes, particularly PDGFRβ-positive ones, after acute ischemic stroke, from tissue repair to functional recovery. First, the vascular mural cells expressing PDGFRβ enhances leptomeningeal arteriogenesis and intrainfarct angiogenesis, thereby promoting recovery of CBF in ischemic areas, which is a prerequisite for tissue repair (Tachibana et al., 2017; Gautam and Yao, 2018). Second, PDGFRβ-mediated repair within infarct areas enhances peri-infarct astrogliosis and oligodendrogenesis, thereby promoting functional recovery (Fig. 9). To the best of our knowledge, this is the first report clearly demonstrating that improved intrainfarct tissue repair may promote functional recovery through the enhancement of astrogliosis and oligodendrogenesis after acute ischemic stroke.

Some clinicians and researchers may consider that post-stroke recovery of CBF within infarct areas is unimportant because there are no neurons that can be rescued after brain infarction is complete. However, leptomeningeal arteriogenesis and intrainfarct angiogenesis certainly occurred (Figs. 2, 3) and contributed to recovery of CBF and subsequent fibrotic formation within infarct areas (Fig. 3). In response to PDGF-BB, PDGFRβ-expressing pericytes/SMCs can produce cytokines and chemokines that recruit monocytes/macrophages. The monocytes/macrophages recruited into the anastomotic vessels induced the vascular remodeling leading to vascular dilation (Scholz et al., 2000; Sugiyama et al., 2011). Because these processes were significantly attenuated in *Pdgfrb^+/–^* mice (Fig. 2), PDGFRβ plays an important role in the formation of arteriogenesis in ischemic areas after pMCAO, a crucial factor for functional recovery (Komori et al., 2019). In addition, PDGFRβ plays crucial roles in microvascular pericytes. Pericytes are very vulnerable and easily detach from endothelial cells after ischemic insult (Tachibana et al., 2017), and thus they have to be re-recruited around endothelial tubes through PDGFRβ to stabilize microvascular structure and to maintain blood flow (Hellstrom et al., 1999; Daneman et al., 2010). PDGFRβ-expressing pericytes transdifferentiate into fibrotic cells and occupy infarct areas to complete the repair while producing extracellular matrix proteins (Makihara et al., 2015). Collectively, pericytes and SMCs may play crucial roles in arteriogenesis, angiogenesis and fibrosis after ischemic stroke through PDGFRβ.

It has been controversial whether the fibrotic tissue repair within injured areas in the CNS is beneficial or not, because it may hinder the chance of neurogenesis and axonal regeneration, particularly after spinal cord injury (Dias and Göritz, 2018; Gautam and Yao, 2018). However, the present study clearly demonstrated that PDGFRβ-mediated fibrotic repair correlated with better functional recovery during the subacute phase after ischemic stroke (Fig. 7). The benefits of postinjury fibrotic repair may be different between the brain and the spinal cord or be dependent upon the timing and/or extent of tissue damage (Adams and Gallo, 2018). A possible mechanism underlying the positive association between intrainfarct repair and functional recovery may be that PDGFRβ-positive pericytes or pericyte-derived cells have gene expression profiles similar to those of mesenchymal stem cells and create a regenerative microenvironment in intrainfarct and peri-infarct areas in the brain while producing various neurotrophic factors, such as BDNF and NT3, and immunomodulatory molecules (Ishitsuka et al., 2012; Chen et al., 2014; Caplan, 2017).

In the present study, defective intrainfarct repair by genetic manipulation of *Pdgfrb* significantly attenuated peri-infarct astrogliosis and oligodendrogenesis, but not neurogenesis. As reported previously, there may be a close association between PDGFRβ-expressing cells and GFAP-positive reactive astrocytes through direct contact with one another at the boundary of brain infarction (Shen et al., 2012; Wanner et al., 2013; Makihara et al., 2015). Based on the present results, we consider that intrainfarct PDGFRβ-positive cells positively regulate astrocyte activity by producing humoral factors (Fig. 4). Among possible factors produced by PDGFRβ-positive cells, we focused on IL6 because it can be produced from mesenchymal cells, including pericytes (Gaceb et al., 2018), and can induce the phosphorylation of STAT3 and AKT, both potent intracellular transducers of activation and survival/proliferation of astrocytes (Yamashita et al., 2005; Nakamura et al., 2012; Wanner et al., 2013; Gu et al., 2016). It is also reported that IL6 can induce post-stroke angiogenesis and repair (Gertz et al., 2012). Indeed, local production of IL6 by pericytes was significantly increased on 7 and 14 d after pMCAO and was significantly attenuated in *Pdgfrb^+/–^* mice. Furthermore, PDGF-BB increased the production of IL6 in cultured PDGFRβ-positive pericytes, while PCM-induced proliferation of cultured astrocytes was attenuated by treatment with an anti-IL6 antibody. Thus, humoral factors produced by PDGFRβ-positive cells, including IL6, may promote astrocyte activation and survival/proliferation in peri-infarct areas (Zamanian et al., 2012).

The most important and interesting finding of this study is that PDGFRβ-mediated intrainfarct repair enhanced peri-infarct oligodendrogenesis as well as astrogliosis (Fig. 5). Oligodendrogenesis consists of three main steps, OPC proliferation, OPC differentiation and myelination (Franklin and Ffrench-Constant, 2008; Marin and Carmichael, 2019). Post-stroke OPC proliferation, as assessed by OLIG2-positive cell number, was promptly induced in peri-infarct areas within 7 d, regardless of intrainfarct tissue repair (Fig. 5). Although we could not identify the specific factors promoting the proliferation of OPCs, demyelination or ischemic insult in the brain may stimulate the proliferation (Franklin and Ffrench-Constant, 2008; Itoh et al., 2015; Marin and Carmichael, 2019). However, the subsequent OPC differentiation and myelination may require significant levels of nutrients and/or trophic factors in an appropriate environment, probably astrogliosis (Burda and Sofroniew, 2014; Cregg et al., 2014; Miyamoto et al., 2015). Consistent with this *in vivo* observation, ACM could induce the differentiation of OPCs and the production of myelinating proteins by cultured OPCs (Fig. 6). Because these responses were enhanced further by the addition of PDGF-BB-stimulated PCM to ACM (Fig. 6), we assume that peri-infarct reactive astrocytes, which are activated by PDGFRβ-expressing cells within infarct areas, are primarily responsible for the OPC differentiation and remyelination. However, we could not exclude the possibility that molecules produced from PDGFRβ-expressing cells directly stimulate OPC differentiation without astrocyte responses, as shown in Extended Data Figure 6-1 and in non-stroke demyelinating animal models (De La Fuente et al., 2017; Montagne et al., 2018). The post-stroke intercellular interaction among pericytes-astrocytes-OLs/OPCs may be essential to complete efficient remyelination in the brain. This may be quite similar to the developmental myelination process: astrogenesis and oligodendrogenesis occur sequentially after the recruitment of PDGFRβ-positive pericytes into endothelial tubes (Daneman et al., 2010). We believe that the concept of repair-induced OPC differentiation and remyelination is very important and a feasible therapeutic strategy for promoting functional recovery after acute ischemic stroke.

There are some limitations in the present study. First, we used conventional *Pdgfrb^+/–^* mice. PDGFRβ is exclusively expressed in pericytes in mice, particularly after ischemic stroke (Bell et al., 2010; Winkler et al., 2010; Arimura et al., 2012; Makihara et al., 2015); therefore, we considered that the effects of PDGFRβ heterozygous deletion after ischemic stroke were attributable mainly to impaired function of pericytes. However, we cannot completely exclude the possibility that PDGFRβ might be expressed in other cell types, such as neurons, astrocytes, and OPCs, and play a role in post-stroke neurorestoration (Shen et al., 2012). Second, because we used the mice with systemic deletion of *Pdgfrb*, there remains a possibility that PDGFRβ-expressing cells in non-CNS organs produced some trophic factors in response to stroke, thereby affecting post-stroke recovery as remote effects. The finding that post-stroke body weight loss was greater in *Pdgfrb^+/–^* mice (Figs. 1-3) may be one of the systemic effects of heterozygous deletion of *Pdgfrb*. Third, we may need to consider other cell types, such as endothelial cells (Zhou et al., 2019), macrophages (Cantuti-Castelvetri et al., 2018) and regulatory T cells (Dombrowski et al., 2017) for their contribution to enhanced oligodendrogenesis after ischemic stroke, as has been shown in demyelinating disorders, although there is a possibility that these cells function cooperatively with pericytes/SMCs and astrocytes to promote oligodendrogenesis. Fourth, although PDGFRβ-mediated intrainfarct repair did not directly affect the number of newly generated neurons in peri-infarct areas (Fig. 5), the possibility remains that it might enhance axonal sprouting of survived or migrating neurons to form new inter-neuron connections in peri-infarct areas, thereby promoting functional recovery (Carmichael et al., 2017). Even so, remyelination is absolutely needed for these neurons to be functional. Fifth, Su et al. (2008) showed that simultaneous administration of imatinib, a PDGFR inhibitor, and recombinant tissue plasminogen activator reduces vascular permeability and hemorrhagic complications in an ischemia-reperfusion animal model (Su et al., 2008). We should note that the significance of PDGFRβ expression may be different between pMCAO and ischemia/reperfusion tMCAO models because post-stroke development of leptomeningeal arteriogenesis and intrainfarct angiogenesis are not always necessary in ischemia-reperfusion stroke models in contrast to pMCAO (Tachibana et al., 2017; Komori et al., 2019). In addition, Su et al. (2008) only evaluated cerebrovascular permeability and hemorrhage 1 d after reperfusion, but not functional recovery in the subacute phase (Su et al., 2008). Finally, although we demonstrated the importance of intercellular interaction among pericytes, astrocytes, and OPCs/OLs in tissue repair and functional recovery, we could not identify the decisive factors responsible for the interaction in this study. We should identify the factors mediating the interaction that leads to functional recovery in future.

In conclusion, we demonstrate that improved tissue repair within infarct areas can bring about better functional recovery through the enhancement of astrogliosis and oligodendrogenesis during the subacute phase. We speculate that post-stroke oligodendrogenesis leading to functional recovery can occur in an appropriate environment, with sufficient blood supply and the presence of active astrocytes. We suggest that promotion of repair is a promising therapeutic regenerative medicine strategy after acute ischemic stroke even considering the limited therapeutic time window.

## Synthesis

Reviewing Editor: Dritan Agalliu, Columbia University Medical Center Decisions are customarily a result of the Reviewing Editor and the peer reviewers coming together and discussing their recommendations until a consensus is reached. When revisions are invited, a fact-based synthesis statement explaining their decision and outlining what is needed to prepare a revision will be listed below. The following reviewer(s) agreed to reveal their identity: Zsuzsa Fabry. Note: If this manuscript was transferred from JNeurosci and a decision was made to accept the manuscript without peer review, a brief statement to this effect will instead be what is listed below.

The topic is important as we still lack knowledge regarding post-stroke tissue regeneration and its connection to functional recovery. These studies characterizing the roles of PDGFRb after ischemic stroke are only moderately innovative and it is unclear whether they provide any novel information.

Post-stroke neurogenesis, astrogliosis and oligodendrogenesis have been studied previously. Several factors have been described, including PDGF-BB that are crucial in promoting post-stroke fibrotic repair in and around the infarct areas, and the role of PDGFBB-PDGFRb has been extensively studied. The authors propose that PDGFRb-mediated activation of pericytes results in an unknown trophic factor/s production that contribute(s) to repair, by regulating peri-infarct astrogliosis, OPC proliferation and remyelination, leptomeningeal arteriogenesis and intra-infarct angiogenesis. Peri-infarct astrogliosis in the PDGFRb +/- animal model was also associated with decreased STAT3 phosphorylation that could be compensated with pericyte-conditioned culture supernatants in vitro cultures of astrocytes. Interestingly, astrocyte conditioned medium promoted OPC differentiation that was enhanced with the addition of pericyte-conditioned medium. Despite the solid data, there are some minor concerns raised by both reviewers of the study that the authors need to address in a revised manuscript.

Reviewer # 1

The experiments are extensive, data heavy and provide a complex picture of post-stroke tissue regeneration. Different aspects of post-stroke tissue recovery are studied by the authors using an array of methodologies including pMCAO, IHC, rtPCR, in vitro cell proliferation and behavior assays. In general, experiments are well designed and straightforward. Visual abstract provided in Figure 8 adequately illustrates the complexity of the post-stroke tissue regeneration process.

However, there are some concerns about the study:

1) While the complex cellular communication between PDGFBB/PDGFRb activated pericytes, astrocytes and OPCs is interesting, without the identification of responsible factors, these studies remain descriptive, rather than mechanistic. Studies presented in Fig 4 showing higher IL6 production in wild type mice on day 7 and 14 after pMCAO are not conclusive.

2) Mice deficient in PDGFb might have additional renal, cardiovascular and hematological abnormalities. Although the authors used +/- mice and show 80% reduction of baseline PDGFRb expression (Fig1-1), the non-CNS phenotype of these mice should still be discussed in the context of ischemic stroke.

3) According to Winkler et al. (Mol. Neurodegeneration 2010), PDGFRb is exclusively expressed in pericytes, and not on neurons, astrocytes or endothelial cells in the adult mouse brain. The authors show Pdgfrb mRNA in neurons and astrocytes. Why is there a discrepancy?

4) PDGFRb signaling deficient mice with progressive pericyte loss have significant BBB leakage, which is more pronounced in the cortex, the primary site of infarct in this model. Increased BBB breakdown as it is also shown in Figure 3F should lead to elevated infarct size. This seems to be the opposite of the proposed hypothesis. The authors need to discuss this issue in detail in the Discussion.

5) Since biological variations in sexes significantly influence infarct outcome in the pMCAO model, justification for using male and female mice (extended figures) should be included.

6) Differences shown in figures 1B, 1C and 1D are difficult to appreciate due to the small differences between group values and sizes for the wild-type and Pdgfrb+/- mice. Therefore, the authors should be cautious when discussing these data.

7) IBA1 is not an exclusive microglia marker (It is also expressed by macrophages). Thus statements that “microglia localized largely in peri-infarct area” should be revised to reflect this issue.

8) SHAM control group should be included in all groups, not just for the functional studies (Fig. 7).

Reviewer # 2

Minor concerns:

1) It is not clear to me what is the message of the study. As already pointed out and discussed by authors - no cell type specific deletion of PDGFRb was performed and therefore the data interpretation is difficult. The authors need to acknowledge and discuss this in the Discussion.

2) The authors discuss the PDGFRB signaling in general. However, they should discuss their results in context of pericytes, vascular smooth cells and astrocytes. The introduction does not give the necessary background and reasons why it is interesting/necessary to study the role of PDGFRB in ischemic stroke. I recommend to rewrite the introduction and discussion for clarity to improve the logic of the study.
