# Supplementary material for: Pericyte-Mediated Tissue Repair through PDGFRβ Promotes Peri-Infarct Astrogliosis, Oligodendrogenesis, and Functional Recovery after Acute Ischemic Stroke
Source: eNeuro. 2020 Mar 3;7(2):ENEURO.0474-19.2020. doi: 10.1523/ENEURO.0474-19.2020 (PMC7070447; doi:10.1523/ENEURO.0474-19.2020)
Supplement: Extended Data Figure 1-1 — Baseline expression of PDGFRβ is decreased in the brain in Pdgfrb+/– mice. Immunoblot analysis demonstrates the baseline decrease in PDGFRβ expression by ∼80% in the brain in Pdgfrb+/– mice compared with wild-type mice (n = 4 mice per group). β-Actin is used as a loading control. Data represent the mean ± SEM (***p < 0.001, unpaired t test). Download Figure 1-1, DOCX file. [file enu-eN-TNC-0474-19-s02.docx]

**Extended Figures**

**
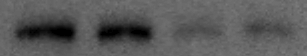

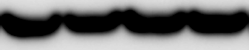
**

PDGFRβ

β-actin

Wild-type

*Pdgfrb^+/-^*

***

PDGFRβ / β-actin

Wild-type

*Pdgfrb^+/-^*

**Figure 1-1. Baseline expression of PDGFRβ is decreased in the brain in *Pdgfrb^+/–^* mice.** Immunoblot analysis demonstrates the baseline decrease in PDGFRβ expression by ~80% in the brain in *Pdgfrb^+/–^* mice compared with wild-type mice (n=4 mice per group). β-actin is used as a loading control. Data represent the mean ± SEM (****p* < 0.001, unpaired t test).

**
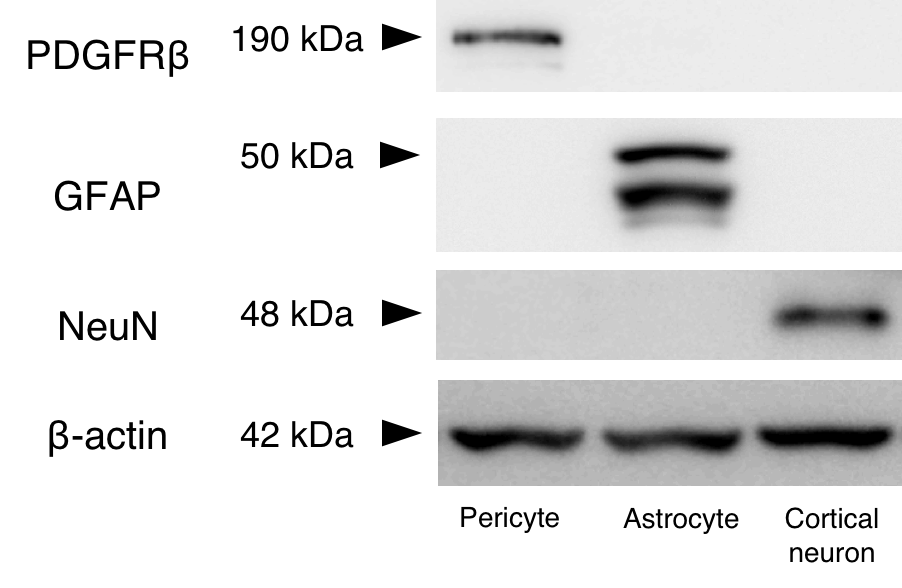
**

**Figure 1-2. Immunoblot analyses of PDGFRβ and cell-specific genes in cultured cells.**

Immunoblot analyses of PDGFRβ, GFAP, NeuN and β-actin in cultured pericytes, astrocytes and cortical neurons. Primary cortical neurons were isolated from ICR mice at embryonic day 17 (E17) as described previously (Nakashima et al., 2018).

**References**

Nakashima H, Tsujimura K, Irie K, Ishizu M, Pan M, Kameda T, Nakashima K (2018) Canonical TGF-β Signaling Negatively Regulates Neuronal Morphogenesis through TGIF/Smad Complex-Mediated CRMP2 Suppression. J Neurosci 38:4791-4810.


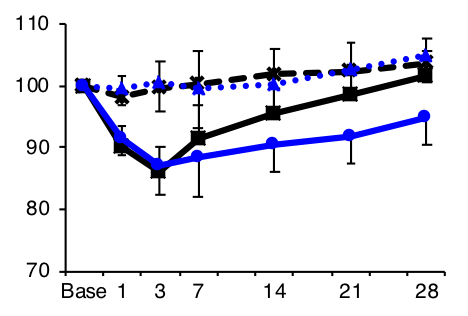


Body weight

(%)

***

***

**

Days

Wild-type

*Pdgfrb^+/-^*

†

**Figure 1-3. Body weight recovery after pMCAO is suppressed in *Pdgfrb^+/–^* mice.** The graph shows body weight changes in wild-type and *Pdgfrb^+/–^* mice at the indicated days after pMCAO (n=12, each group). Data represent the mean ± SEM (^†^*p* < 0.1, ***p* < 0.01 and ****p* < 0.001, unpaired t test).


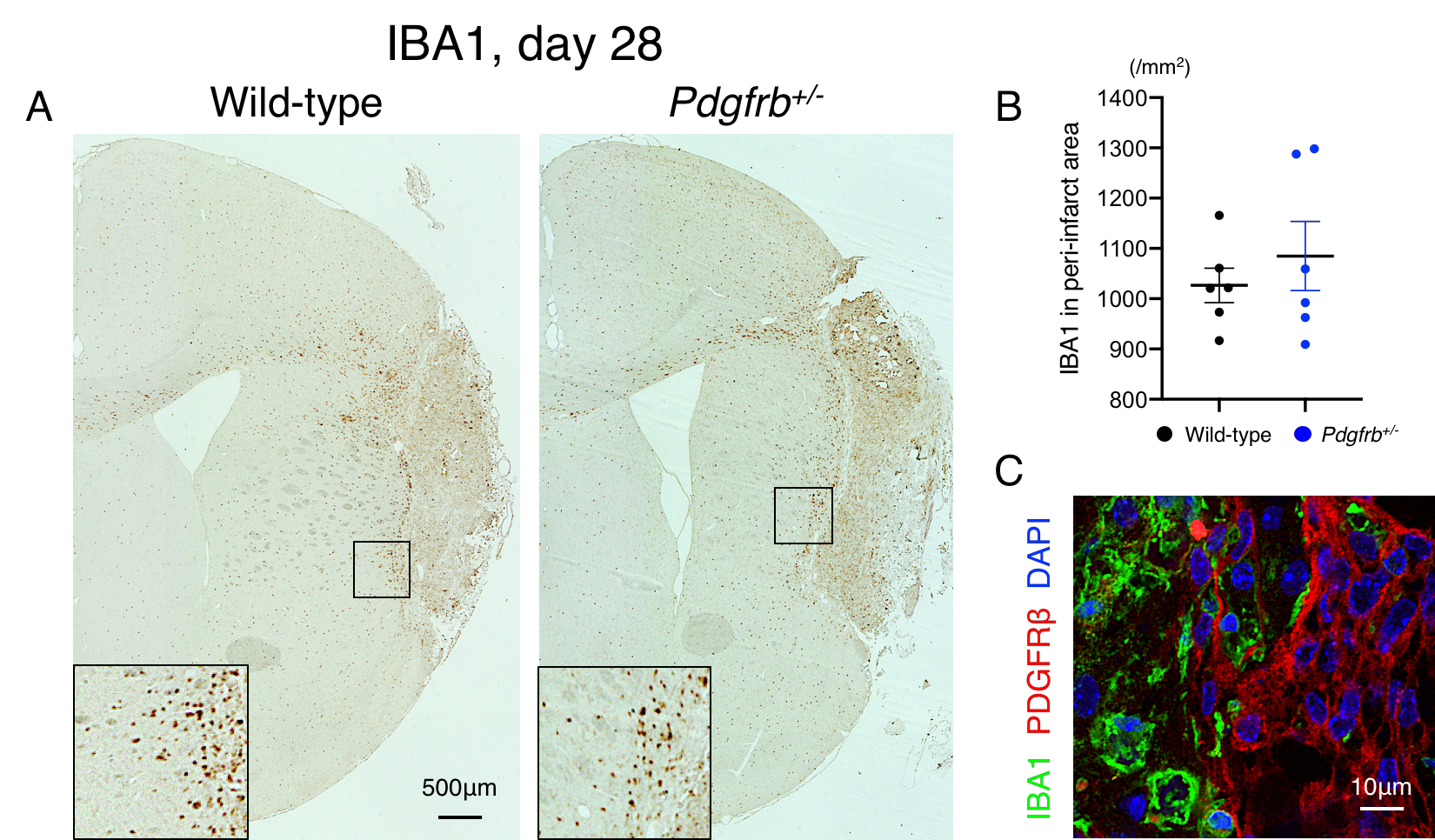


**Figure 4-1. Localization and extent of IBA1-positive microglia/macrophage on day 28 after pMCAO.** (A) Representative IBA1 staining on day 28 after pMCAO in wild-type (left) and *Pdgfrb^+/–^* (right) mice. Scale bar, 500 μm. (B) Quantification of the number of IBA1-positive cells in peri-infarct areas on day 28 after pMCAO in wild-type (black) and *Pdgfrb^+/–^* mice (blue) (n=6, each group; p=0.465, unpaired t test). (C) Representative immunofluorescence labeling for IBA1 (green) and PDGFRβ (red), and DAPI (blue) in peri-infarct area on day 28 after pMCAO in wild-type mice (Scale bar, 10 μm). Data represent the mean ± SEM.


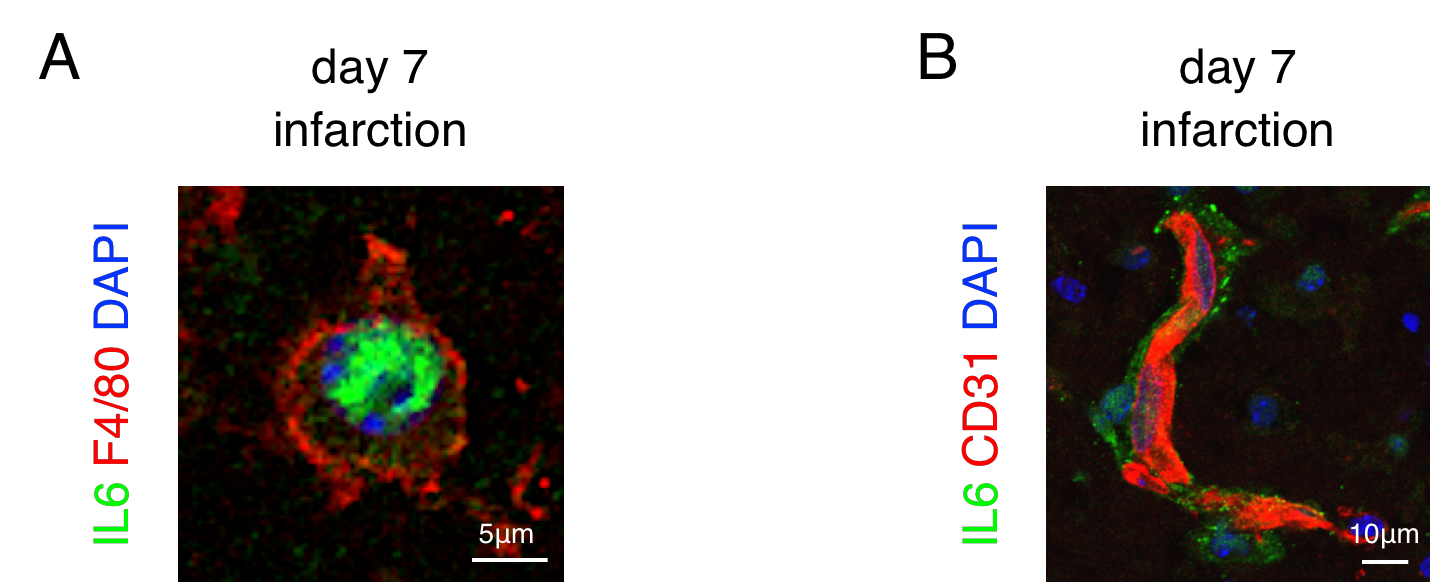


**Figure 4-2. IL6 expression within infarct area after pMCAO.** Double immunofluorescence labeling of and IL6 (green) and (A) F4/80 (red), a marker of macrophage (scale bar, 5 μm), or (B) CD31 (red), a marker of endothelial cell (scale bar, 10 μm), on day 7 after pMCAO.

**
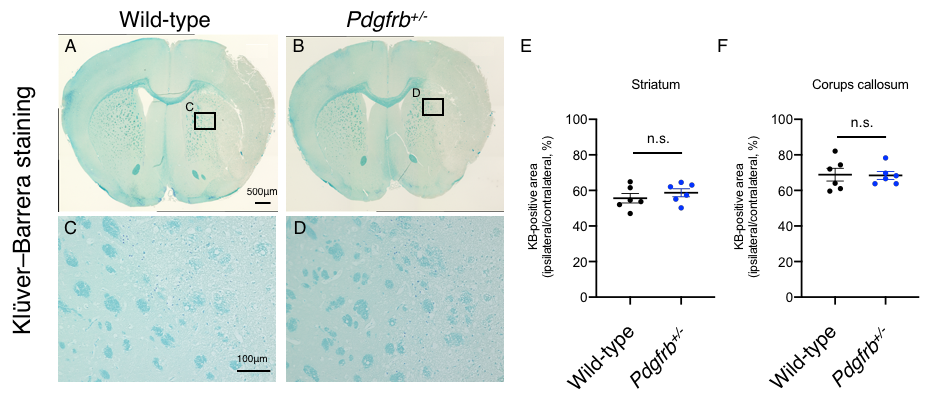
**

**Figure 5-1. White matter injury after pMCAO is comparable between wild-type and *Pdgfrb^+/–^*** **mice.** (A, B) Representative Klüver–Barrera (KB) staining on day 1 after pMCAO in wild-type and *Pdgfrb^+/–^* mice. Scale bar, 500 μm. (C, D) Magnified images of peri-infarct striatum are shown. Scale bar, 100 μm. (E, F) The extent of white matter injury is assessed by KB-positive area in striatum and corpus callosum against that of the contralateral hemisphere (n=6, each group, unpaired t test). Data represent the mean ± SEM.

**
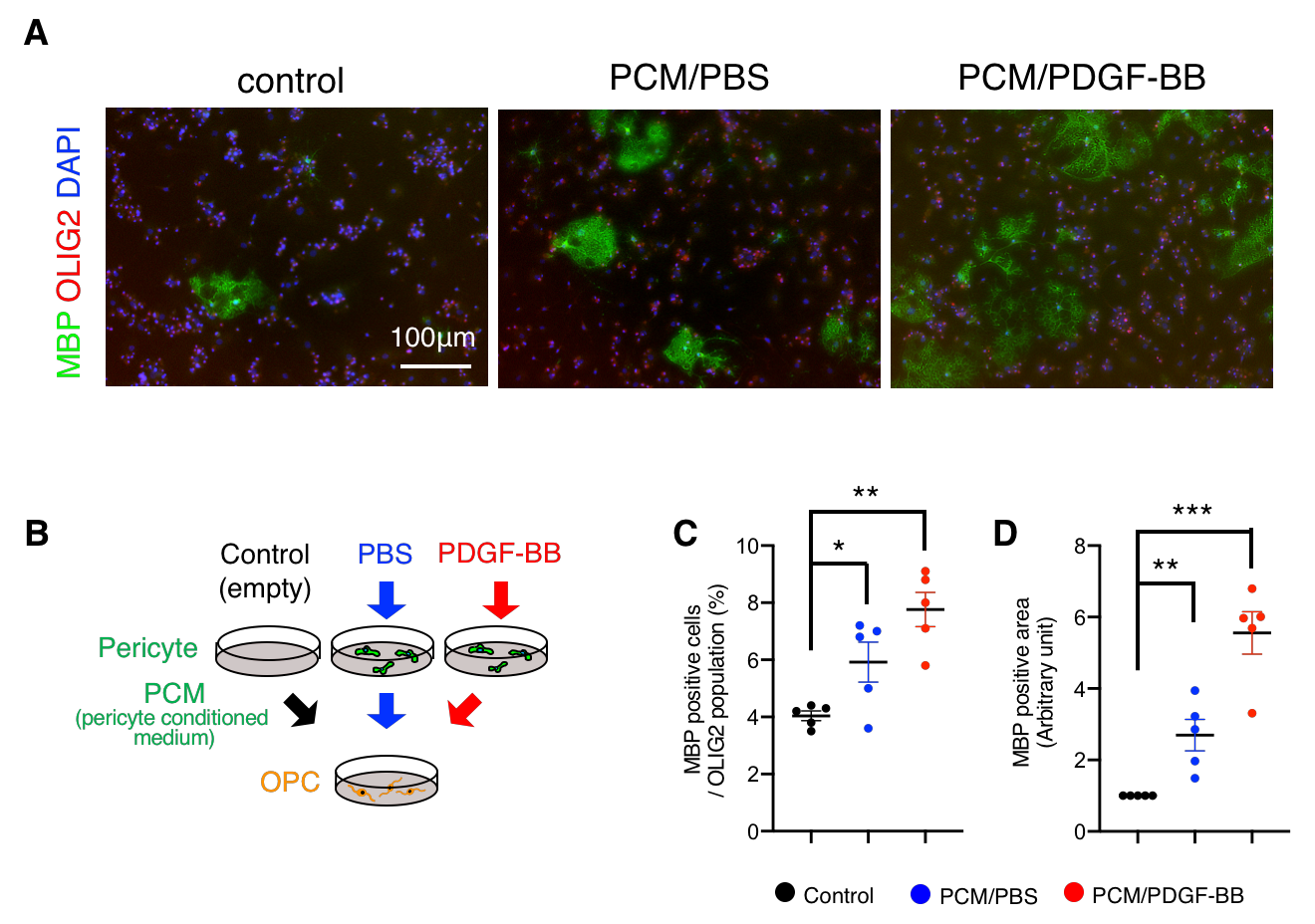
**

**Figure 6-1. Effects of pericyte-conditioned medium on OPC differentiation and myelination.** (A) Double immunofluorescence labeling of MBP (green) and OLIG2 (red) in cultured OPC treated with pericyte-conditioned medium (PCM) for 7 days (n= 5, each group) (scale bar, 100 μm). (B) An experimental scheme for oligodendrocyte precursor cell (OPC) differentiation in normal culture medium (control) or in PCM treated with PBS (blue, PCM/PBS) or PDGF-BB (10 ng/ml) (red, PCM/PDGF-BB). (C, D) Quantification of the number of MBP-positive cells within OLIG2-positive OPCs and MBP-positive areas. Data represent the mean ± SEM (**p* < 0.05, ***p* < 0.01, and ****p* < 0.001 one-way ANOVA followed by Bonferroni's post-test).
